# Facile metabolic reprogramming distinguishes mycobacterial adaptation to hypoxia and starvation: ketosis drives starvation-induced persistence in *M. bovis* BCG

**DOI:** 10.1038/s42003-024-06562-2

**Published:** 2024-07-16

**Authors:** Nick K. Davis, Yok Hian Chionh, Megan E. McBee, Fabian Hia, Duanduan Ma, Liang Cui, Mariam Lucila Sharaf, Weiling Maggie Cai, Watthanachai Jumpathong, Stuart S. Levine, Sylvie Alonso, Peter C. Dedon

**Affiliations:** 1 Department of Biological Engineering, Massachusetts Institute of Technology, Cambridge, MA USA; 2 Antimicrobial Resistance Interdisciplinary Research Group, Singapore-MIT Alliance for Research and Technology, Singapore, Singapore; 3 Infectious Diseases Translational Research Programme, Department of Microbiology and Immunology, Yong Loo Lin School of Medicine, National University of Singapore, Singapore, Singapore; 4 The David H. Koch Institute for Integrative Cancer Research, Massachusetts Institute of Technology, Cambridge, MA USA; 5 Department of Biology, Massachusetts Institute of Technology, Cambridge, MA USA; 6 Immunology Programme, Life Sciences Institute, National University of Singapore, Singapore, Singapore; 7 Present Address: GenScript Biotech (Singapore) Pte. Ltd, Singapore, Singapore; 8 Present Address: BioNTech SE An der Goldgrube, Mainz, Germany; 9 Present Address: British High Commission, Singapore, Singapore; 10 Present Address: Chemical Biology Program, Chulabhorn Graduate Institute, Bangkok, Thailand

**Keywords:** Pathogens, Biochemical networks, Bacterial pathogenesis

## Abstract

Mycobacteria adapt to infection stresses by entering a reversible non-replicating persistence (NRP) with slow or no cell growth and broad antimicrobial tolerance. Hypoxia and nutrient deprivation are two well-studied stresses commonly used to model the NRP, yet little is known about the molecular differences in mycobacterial adaptation to these distinct stresses that lead to a comparable NRP phenotype. Here we performed a multisystem interrogation of the *Mycobacterium bovis* BCG (BCG) starvation response, which revealed a coordinated metabolic shift away from the glycolysis of nutrient-replete growth to depletion of lipid stores, lipolysis, and fatty acid ß-oxidation in NRP. This contrasts with BCG’s NRP hypoxia response involving a shift to cholesterol metabolism and triglyceride storage. Our analysis reveals cryptic metabolic vulnerabilities of the starvation-induced NRP state, such as their newfound hypersensitivity to H_2_O_2_. These observations pave the way for developing precision therapeutics against these otherwise drug refractory pathogens.

## Abstract (web summary)

A multi-omics comparison of the mycobacterial response to hypoxia and starvation reveals that distinct differences in fatty acid and cholesterol metabolism lead to the same non-replicating, drug-tolerant persistence phenotype.

## Introduction

M*ycobacterium tuberculosis* (Mtb) remains the single most lethal human bacterial pathogen in the world^1^. With humans as its only host, Mtb adapts to the stress of infection by entering a state of non-replicating persistence (NRP) marked by limited cell division, immune quiescence, and increased antibiotic tolerance^2,3^. The molecular mechanisms underlying the transition to and survival in NRP remain poorly defined, however. One clue may lie in the fact that Mtb, in contrast to other bacteria, devote a significant portion of genomic coding capacity to enzymes involved in lipogenesis and lipolysis, with growing evidence that lipid biosynthesis and fatty acid metabolism play key roles in Mtb’s pathogenicity^4,5^. During acute infection, mycobacteria survive by subverting phagolysosome biogenesis and acidification, progressively gaining access to the cytosol and modulating host immune responses^6^. During chronic infection, it is believed that mycobacteria sequester themselves within macrophages, where oxygen and nutrient deprivation trigger the NRP phenotype. While the hypoxic response has been well characterized in members of the Mtb complex, our understanding of how mycobacteria adapt to nutrient starvation remains incomplete^7^.

Starvation of mycobacteria has been shown to cause altered central carbon metabolism^8^, global transcriptional reprogramming^9^, the induction of virulence-enhancing toxin–antitoxin modules^10^, altered permeability to drugs and metabolites^11,12^, and phenotypic resistance to mechanistically diverse antibiotics^13,14^. Despite extensive study, previous investigations of mycobacterial starvation have predominantly relied on monitoring changes in gene expression triggered by growth on single carbon sources^15^, collating phenotypic and essentiality differences among single-gene mutants^16^, and mapping transcription factor binding sites across the genome^17^. The diversity of experimental conditions makes it difficult to compare findings between these studies, and few attempts have been made to compare responses to starvation and hypoxia in the same study, which makes it difficult to define how metabolic adaptations to two very different stresses lead to the same NRP phenotype.

In this work, we describe an exhaustive set of systems-level analyses that all point to unique shifts in lipid metabolism as a feature that distinguishes the mycobacterial response to hypoxia and starvation despite the common NRP endpoint. Here we use unsupervised multivariate analyses to integrate a variety of biochemical parameters, phenotypic metabolic profiling, time-course metabolomics, longitudinal RNA-seq, and quantitative proteomics. These analyses revealed that, in contrast to a hypoxia-induced shift to cholesterol metabolism and triglyceride accumulation, starvation induces a metabolic shift to β-oxidation of fatty acids and a resulting ketotic state. This transition induces a unique vulnerability to H_2_O_2_ that is not observed in hypoxia-induced NRP^18^. Together, these findings underscore the power of integrated systems approaches to understanding how pathogens respond to stress and suggest that the metabolomic heterogeneity of the NRP state could present an exploitable vulnerability for the development of novel TB chemotherapies.

## Results

### Starved mycobacteria exhibit hallmarks of NRP

**Fig. 1:**
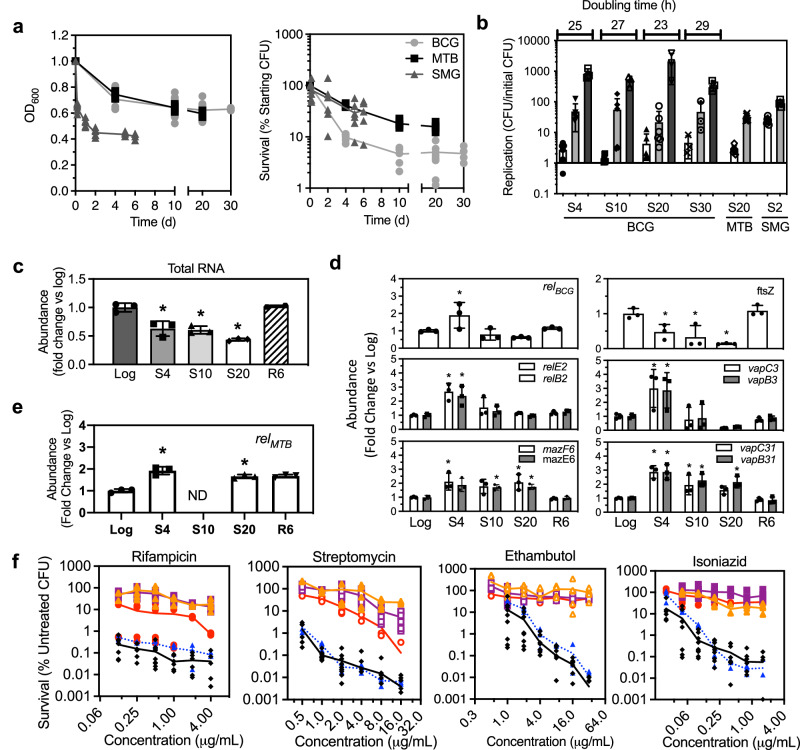
Characteristics of starved mycobacterial persisters. **a** Survival profiles (OD_600_, CFU) of MTB, BCG, and SMG during nutrient deprivation in PBS. **b** Recovery of BCG after 4, 10, 20, or 30 days of starvation in PBS (S4, S10, S20, and S30, respectively) followed by 2 (white), 6 (gray), and 10 days (black) of resuscitation in nutrient-replete media; MTB after 20 days of starvation followed by 2 days (white) and 6 days (gray) of resuscitation; and SMG after 6 days of starvation followed by 9 h (white) and 24 h (black) of resuscitation. Doubling times are not significantly different by one-way ANOVA with post hoc Tukey's HSD. Data are shown as mean ± SE for *n* ≥ 3. **c** Reduction in total cellular RNA levels in BCG at S4, S10 and S20, with restoration at R6. **d** Transcriptional induction of the stringent response associated genes *relBCG*, *relBE2*, *mazEF6*, *vapBC3,* and *vapBC31* in BCG, and repression of cell division marker *ftsZ* measured by RNA-seq. **e** Expression of *relMTB* in BCG measured by qPCR. **f** Antibiotic susceptibility of Log (◆), S4 (), S10 (), S20 (), and R6 () BCG after 48 h of exposure. Data represent mean ± SD for *n* ≥ 6. **c**–**e** Data represent mean ± SD for *n* ≥ 3; *P < 0.05 determined by one-way ANOVA with Dunnett's test vs. Log. See Supplementary Data 4 for the source data used in these graphs.

To initiate our mechanistic analysis, we first established a suitable experimental model for starvation-induced NRP, which was consistent with our previous studies of hypoxia-induced NRP in *Mycobacterium bovis* BCG str. Pasteur 1173P2 (BCG), a member of the Mtb Complex. Consistent with other experimental models of bacterial persistence^19^, nutrient deprivation by culturing *M. tuberculosis* CDC1551 (Mtb), BCG, and *Mycobacterium smegmatis* MC^2^155 in phosphate-buffered saline (PBS) resulted in biphasic death kinetics characterized by a rapid decrease in both OD_600_ and CFU within the first 4 days of starvation (S4), followed by a slower decline to 10–30% of initial CFU (S10-S30 for BCG, Fig. 1a). Using flow cytometry, we observed coalescence of population forward- and side-scatter values in starved BCG cultures (Supplementary Fig. 1a and Supplementary Table 1), suggesting that mycobacteria assume smaller overall cell size and reduced cellular complexity during prolonged nutrient deprivation. These starvation-induced morphological transitions are consistent with previous studies of MTB isolated from lung lesions^6^. Intriguingly, we found divalent cations to be major determinants of mycobacterial survival during starvation, with growth in divalent cation-free PBS (compared to Dulbecco’s PBS containing Mg^2+^, Ca^2+^) required to induce NRP in MTB, BCG, and SMG in vitro (Supplementary Fig. 1b, c). When starved in Mg^2+^- and Ca^2+^-containing DPBS, significantly more BCG and SMG survived compared to starvation in PBS (Supplementary Fig. 1b, c). This detail may explain why previous starvation studies report no loss of MTB viability during growth in Mg^2+^- and Ca^2+^-containing PBS (i.e., Dulbecco’s PBS)^9,14^. We further assessed starved cultures for the possibility that nonviable cells (~95% of initial CFU) served as a carbon source for viable starved BCG persisters in culture. Washing and re-culturing the cells in fresh PBS did not result in increased cell death (Supplementary Fig. 1d), which rules out cannibalism as the basis for NRP, and cells retained their ability to regrow (“resuscitate”) in nutrient-replete 7H9 media (resuscitation day 6 or R6, Fig. 1b). Thus, it appears that neither a transition to a viable but nonculturable state, nor cannibalism alone, accounted for mycobacterial survival over prolonged periods of starvation^20,21^.

After establishing that starved mycobacteria remain viable, we characterized starved BCG for the transcriptional, phenotypic, metabolomic, and proteomic hallmarks of bacterial persistence^3^. Consistent with the observed transcriptional induction of RelA-SpoT homologs (RSH) in the stringent responses of other bacteria^22^, mRNA levels for *rel*_*BCG*_ and *rel*_*MTB*_—genes that control intracellular levels of pp(p)Gpp in BCG and MTB—peaked in early starvation (S4) (Fig. 1d and Supplementary Data 1) and were associated with a concomitant 40% reduction in total RNA content per cell relative to log-growing bacilli (Fig. 1c and Supplementary Figs. 1e and 2a). Total RNA levels decreased at S4, remained low over the course of starvation, and returned to pre-starvation levels upon 6 days of resuscitation in nutrient-replete 7H9 media (R6, Fig. 1c). Similarly, we observed the induction of several important toxin–antitoxin modules (*relBE2*, *mazEF6*, *vapBC3*, *vapBC31*) and reduced expression of the cell division gene *ftsZ* at S4 (Fig. 1d). Starved BCG cultures also developed tolerance against bactericidal doses of the conventional antitubercular antibiotics rifampicin, streptomycin, ethambutol and isonaizid (Fig. 1f). Importantly, the drug-tolerant phenotype was reversed by resuscitation in 7H9 medium, which is consistent with the phenotypic drug tolerance that is a hallmark of NRP^13,14^.

To characterize the molecular changes induced by starvation in BCG, we performed quantitative proteomics to longitudinally profile the time course of NRP in starved BCG, with the detection of 1102 proteins common to three biological replicates in all time points (Supplementary Data 2 and Supplementary Fig. 3a). A principal component analysis (PCA) of the proteomic changes during starvation showed subsets of proteins strongly distinguishing the four time points (Supplementary Fig. 3b), with KEGG pathway enrichment analysis for significantly upregulated (>1.3-fold) proteins underscoring phenotypic differences between early (S4) and late (S20) non-replicating persistence and resuscitation (R6). For example, starvation caused a significant shift away from cell wall anabolism (e.g., CmaA2, EmbB, MmaA2, and GlmU as negative predictors of the starvation response) and toward lipid catabolism (e.g., FabD2, FadE16, GlgE and BCG_0220 as positive predictors of starvation), with the exception of upregulated virulence lipid biogenesis factors phthiocerol dimycocerosates (PDIM; e.g., BCG 2974, PpsB, and PpsD) and mannosyl-β-1-phosphomycoketide (MPM; e.g., Pks12 and Pks13). BCG 2974 is a trans-acting enoyl reductase in the biosynthesis of PDIMs and glycosylated phenolphthiocerol dimycocerosates, which are major cell-surface virulence factors of Mtb during the early macrophage invasion step of infection^23^. KEGG analysis of the proteomics data also identified protein predictors that modulate host immunity (Eis), participate in energy homeostasis (AtpH, CoaE, CtaD, GlgP, PckA, and PgmA), maintain proteome integrity (HtpX, PepC, PepN, PepQ, PpiA2, and PpiB), sequester metal cations (CtpI, HemB and HemC), and, as discussed in detail shortly, serve putative roles in fatty acid β-oxidation (Acs, FadB2, FadB4, FadB5, FadE2, FadE16, FadE23, FadE24, FadE36, EchA8, EchA9, EchA16, FadA, FadA2, and FadA4). The coordinated upregulation of lipid dehydrogenases and oxidoreductases suggests that persisters upregulate fatty acid metabolism during nutrient deprivation. On the other hand, our analysis identified fatty acid biosynthetic enzymes AcpP and FadD26 as strong contravariants of the starvation response, suggesting downregulation of de novo fatty acid synthesis during nutrient deprivation. While oxaloacetate would typically facilitate the integration of Ac-CoA from fatty acid β-oxidation into the tricarboxylic acid (TCA) cycle, our proteomic results indicate the induction of alternative metabolic pathways during starvation. For instance, the general upregulation of thiolase II enzymes (e.g., FadA) suggests increased metabolic flux towards acetoacetate (AcAc)^24^.

These systematic analyses demonstrate that well-controlled nutrient deprivation predictably induces a stable, phenotypically drug-tolerant sub-population in MTB, BCG, and SMG cultures, with BCG serving as a slow-growing experimental surrogate of MTB. While both hypoxia and nutrient deprivation lead to the same drug-tolerant NRP phenotype, it is unclear how mycobacteria adapt to these significantly different stresses. We addressed this issue with a comparative multi-omic analysis.

### Proteomics and transcriptional profiling predict distinct metabolic adaptations in starvation- and hypoxia-induced NRP

**Fig. 2:**
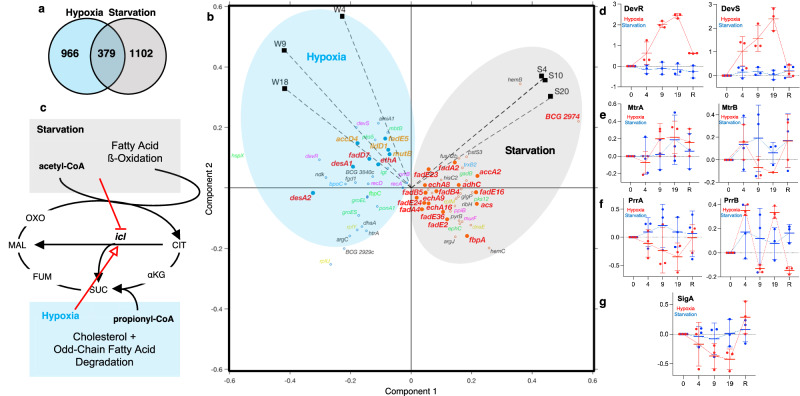
Functional gene responses are different between starvation- and hypoxia-induced NRP. **a** Proteins detected and quantified across time-course proteomic analyses of starvation- (green circle: 1102 proteins) and hypoxia-induced (blue circle: 966 proteins) NRP. A total of 379 proteins were quantified across all time points in both conditions. **b** Principal component analysis reveals distinct protein dynamics during starvation (green ellipse: S4, S10, and S20) and hypoxia (blue ellipse: W4, W9, and W18) responses. Protein observations are color-coded according to the functional category, as follows: red = lipid metabolism (e.g., acetyl-CoA metabolism, β-oxidation); orange = cholesterol metabolism (e.g., propionyl-CoA metabolism); green = cell wall, virulence, detoxification and adaptation; blue = redox homeostasis; magenta = regulatory proteins and information pathways; yellow = replication and translation. **c** Major metabolic differences between starvation- and hypoxia-induced NRP informed by proteomic datasets. While co-catabolism of carbon substrates appears to be a shared feature of both stresses, starvation significantly induces the upregulation of proteins with known functions in fatty acid metabolism. On the other hand, hypoxia coordinately induces the upregulation of proteins with documented roles in the metabolism of cholesterol and odd-chain fatty acids (e.g., enzymes involved in the methylmalonyl-CoA pathway (MCP) and methylcitrate cycle (MCC)).^30^ Importantly, starvation significantly decreased and hypoxia increased levels of isocitrate lyase (Icl), the key enzyme in the glyoxylate shunt. OXO oxaloacetate, CIT citrate, αKG α-ketoglutamate, SUC succinate, FUM fumarate, MAL malate. **d**–**g** Comparison of two-component system protein levels during hypoxia (red) and starvation (blue time courses. Plots contain individual log_2_(fold-change) data for 1–3 replicates, thick bars as mean values and thin bars as standard deviation on 0, 4, 9, and 19 days of hypoxia or starvation and after 6 days of resuscitation (R) with normoxia or nutrient restoration. Note: peptide signals for MtrB in hypoxia did not meet the cutoff criteria for proteomic quantitation but we have added the data in (**e**) for completeness. See Supplementary Data 4 for the source data used in these graphs.

Here, we sought to discern how the functional expression of mycobacterial genes differed between the BCG model for starvation-induced NRP and an established BCG in vitro model for hypoxia-induced NRP^25^. We first compared the proteomics data for the BCG starvation time course (1102 proteins) with our previous analysis of the hypoxic BCG proteome^26^ (966 proteins; Supplementary Data 2 and Fig. 2a). The correspondence between the two stress time courses is as follows: exponentially growing bacilli (Log), early adaptive NRP response (NRP1; day 4 of starvation, S4; day 4 of hypoxia, W4), enduring NRP response (NRP2; days 10 and 20 of starvation, S10 and S20; days 9 and 18 of hypoxia, W9 and W18), and resuscitation (day 6 of nutrient restoration, S-R6; day 6 of normoxia, W-R6). We identified 379 proteins that had quantitative coverage across all 5 related time points in both hypoxia and starvation proteomic datasets (Supplementary Data 2 and Fig. 2a). The two datasets differ strikingly in several respects.

First, a survey of transcriptional regulators reveals divergent pathways of gene expression expected for the response to the two distinct stressors of hypoxia and starvation. For instance, one of the most upregulated genes for hypoxia-induced persisters, at both the transcript and protein levels, is the chaperone HspX^26,27^, which, surprisingly, is one of the most downregulated genes terms of mRNA and protein abundances for starvation-induced NRP (Supplementary Data 1 and 2). *hspX* is under the control of DevSR, which suggests a divergence of transcriptional responses resulting in a phenotypically similar persistent state. Indeed, as shown in Fig. 2d–g, six of seven transcription factors detected in both stress conditions were differentially regulated. The two-component (TC) system that regulates the early hypoxic response, DevRS, confirms the quantitative rigor of the proteomics analyses, with an expected large increase in both DevR and DevS in hypoxia and no changes during starvation (Fig. 2d). While there is a claim for an essential interaction between the housekeeping sigma factor SigA and DevR^24^, SigA levels decreased over the hypoxia time course and were unchanged in starvation (Fig. 2g). The parallel reduction of members of another TC system, PrrAB, in hypoxia but not in starvation (Fig. 2f) is consistent with Prr function in regulating respiratory and oxidative phosphorylation pathways^28^, as discussed shortly. The small increases in MtrAB TC proteins for both hypoxia and starvation (Fig. 2e) could reflect the role of this system in cell division and cell wall metabolism given the common NRP endpoint of no or slow growth^29^. Another example of the divergence of gene expression for hypoxia and starvation involves biosynthesis and transport of the phthiocerol dimycocerosate (PDIM) cell wall lipid. While starvation upregulated PDIM biosynthesis pathways and the PDIM membrane transporter MmpL7 (Supplementary Fig. 4a and Supplementary Data 2), hypoxia strongly reduced MmpL7 and PDIM synthesis enzymes in hypoxia (Supplementary Fig. 4a and Supplementary Data 2). These observations demonstrate similarities and differences in protein levels for hypoxia and starvation for a variety of BCG metabolic pathways, with parallel observations with metabolites discussed shortly.

Despite some strong similarities in the broad KEGG pathways associated with these 379 proteins during the hypoxic and starvation stresses (Supplementary Fig. 3c–e), PCA of covarying proteins for the two stresses showed significant differences between hypoxia and starvation (Fig. 2b). Among the most striking differences was the starvation-induced upregulation of enzymes involved in fatty acid β-oxidation: acetyl coenzyme A synthetase (Acs), long-chain fatty acyl-CoA ligase (BCG 2974), NADPH oxidoreductase (FadB4, FadB5), acyl-CoA dehydrogenase (FadE2, FadE16, FadE23, FadE24, FadE36), enoyl-CoA hydratase (EchA8, EchA9, EchA16), and acyl-CoA thiolase (FadA, FadA2, and FadA4) (Supplementary Data 2 and Fig. 2c). The coordinated increase in lipid dehydrogenases and oxidoreductases suggests that persisters upregulate fatty acid metabolism during nutrient deprivation. In contrast, PCA showed that one of the strongest hallmarks of the hypoxia response was the upregulation of cholesterol and odd-chain fatty acid metabolism and a strong theme of cell wall biogenesis: upregulated propionyl-CoA decarboxylase (AccD4), acyl-[acyl-carrier protein] desaturase (DesA1 and DesA2), acyl-CoA dehydrogenase (FadE4 and FadE5), l-lactate dehydrogenase (LldD1), methylmalonyl-CoA (MutB), 3-ketoacyl-ACP reductase (FabG and FabG2), monooxygenase (EthA), isocitrate lyase (Icl), citrate synthase (GltA), diacylglycerol O-acyltransferase (BCG_3794c), and essential cell wall formation proteins (EmbA, Lgt and PonA1) (Supplementary Data 2 and Fig. 2c). Cholesterol catabolism provides a carbon source for energy production and restructuring of cell wall lipids, and has been extensively characterized in mycobacteria^8^. Cholesterol is broken down to acetyl-CoA for the TCA cycle, propionyl-CoA for the methylcitrate cycle, the vitamin B12-dependent methylmalonyl pathway or lipid synthesis (e.g., triacylglyceride and PDIM formation), and pyruvate for the generation of acetyl-CoA or to fuel gluconeogenesis. Upregulated LldD1 drives metabolic flux towards pyruvate, upregulated MutB facilitates the synthesis of propionate from TCA cycle intermediates, and upregulated GltA accelerates carbon assimilation into the TCA cycle by facilitating the condensation of acetyl-CoA and oxaloacetate to form citrate (the first reaction of the TCA cycle). These proteomic signals align with cholesterol and odd-chain fatty acid metabolism (Fig. 2b, c): MutB facilitates the generation of 3-carbon intermediates in the form of methylmalonyl-CoA that can be used as building blocks for mycobacterial cell wall lipids, and the upregulation of FabG, FabG2, and BCG_3794c, which participate in TAG biosynthesis, agrees with the observation that mycobacteria increase lipid deposition during the early stages of NRP in macrophages^8,15,30^.

**Fig. 3:**
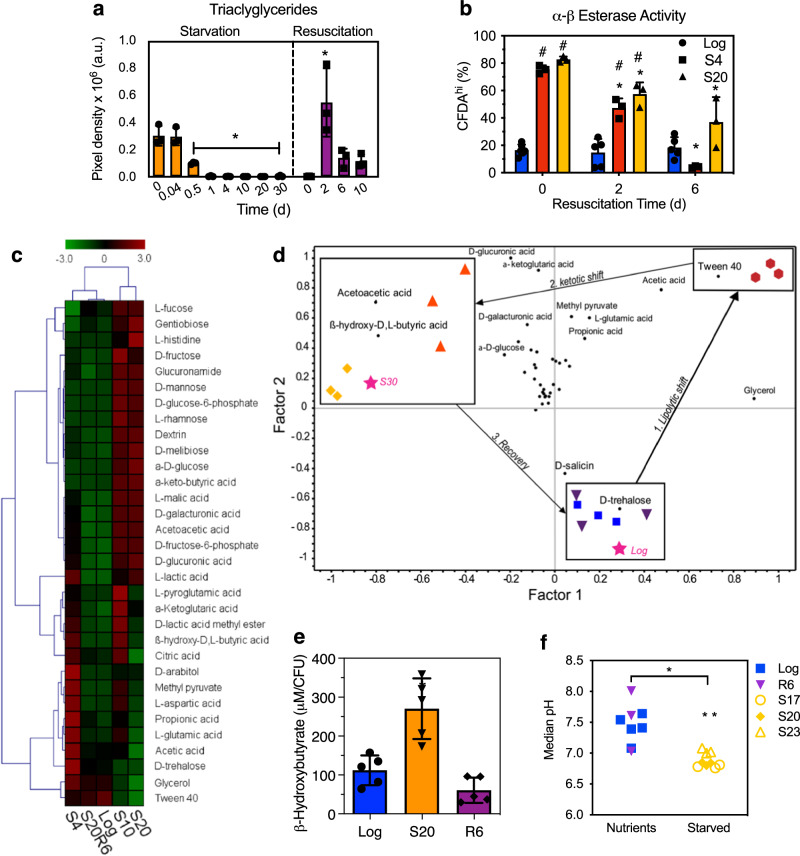
Starvation induces shifts in lipid and ketone body metabolism. **a** TAG content of BCG analyzed by densitometry of thin-layer chromatograms. *N* = 3; *P < 0.05; one-way ANOVA with Dunnett's test vs day 0. **b** Percentage of cells with elevated esterase activity (CFDA^hi^) during starvation and resuscitation. *N* = 6; two-way ANOVA with Bonferroni post tests, ^#^*P* < 0.05 vs log; *P < 0.05 vs resuscitation day 0. **c** Hierarchical clustering analysis of the metabolic phenotype of Log, S4, S10, S20, and R6 BCG on carbon sources that induced growth. Heatmap denotes signals from tetrazolium dye reduction, reflecting carbon utilization relative to the positive control. **d** PCA bi-plot of PLS-DA scores and loadings of metabolic phenotype datasets (*n* = 3 per condition) based on carbon sources with significant dye reduction. Statistical analysis: one-way ANOVA with Bonferroni post test. The PLS-DA model is based on correlation coefficients between PCA scores (condition) and loadings (metabolite utilization) whereby proximity of the carbon source to the condition characterizes the condition. These predictors were used to successfully differentiate independent Log and S30 samples. **e** Intracellular β-hydroxybutyrat**e** in Log, S20, and R6 cultures. *N* = 6; *P < 0.05; one-way ANOVA with Dunnett's test vs Log. (**f**) pH of BCG with nutrients or starved in PBS. Each symbol denotes the median pH of >50,000 cells; *P < 0.05; unpaired two-tailed *t* test with Welch’s correction. See Supplementary Data 4 for the source data used in these graphs.

The metabolic divergence in hypoxia and starvation is reinforced by analysis of fatty acid metabolism. While hypoxia causes accumulation of lipid droplets^31^, we observed starvation-induced depletion of intracellular triacylglycerol stores within just one day of starvation (Fig. 3a and Supplementary Fig. 5a) associated with increased α,β-esterase activity (Fig. 3b and Supplementary Fig. 5b–h), an established proxy for lipase activity in mycobacteria^32^, with LipY being the main lipase for the hydrolysis of triacylglycerols^33,34^. Further, both transcriptomics and proteomics data show that hypoxia induces a general reduction in the levels of enzymes involved in fatty acid β-oxidation and synthesis of long-chain mycolic acids (Supplementary Data 1 and 2). Importantly, the hallmarks of β-oxidation observed during starvation uniformly reversed upon resuscitation (Fig. 3a, b and Supplementary Fig. 5a–h). Collectively, these results suggest that starved persisters actively reprogram their metabolism toward fatty acid β-oxidation, a finding that concurs with previous observations of in vitro stress and in vivo survival^6,9,30,35^, while hypoxic mycobacteria upregulate pathways associated with both cholesterol and odd-chain fatty acid metabolism. Despite the same common endpoint of NRP, these distinct metabolic shifts have different consequences for the cell, including starvation-induced ketosis resulting from substantial increases in β-oxidation of fatty acids during starvation, which we explored next.

### Starvation induces a ketotic metabolic state in mycobacterial persisters

The data to this point predict a starvation-induced metabolic shift to increased β-oxidation of fatty acids, which should lead to downregulation of glucose metabolism and a ketotic state caused by the buildup of ketone bodies such as AcAc and β-hydroxybutyrate (BHB). To test these predictions, we used phenotypic profiling and metabolomics to define the metabolic shifts that mediate mycobacterial persistence during the BCG starvation time course. Phenotypic profiling involved incubating starved BCG on an array of 71 different metabolites, of which 41 induced metabolic activity (tetrazolium dye reduction) and 32 induced cell growth (recoverable CFU) (Supplementary Tables 2 and 3). Unsupervised hierarchical clustering of signals from tetrazolium reduction during culture on the 32 growth-enabling compounds clearly distinguished exponential growth in nutrient-rich media (Log and R6) from both early (S4) and late starvation (S10 and S20) (Fig. 3c). Given the observed metabolic distinctions between starved and exponentially growing BCG, we sought to discriminate carbon source preferences at each timepoint of the starvation experiment. Here analysis of metabolic phenotypes using partial least squares regression (PLSR) revealed a strong correlation between Tween 40 metabolism and early starvation (S4), and between metabolism of ketone bodies (AcAc, BHB) and late starvation (S10 and S20) (Fig. 3d and Supplementary Table 4). The latter was unexpected and suggested that nutrient-deprived persisters may have an increased capacity to metabolize end-products of fatty acid β-oxidation. While the chemical pathology of these metabolites is well-documented in human diabetes^36^, the generation and fate of ketone bodies in bacterial pathogens has not been studied. The presence of a ketotic state in starved BCG was evident in increased levels of BHB (Fig. 3e) and lower intracellular pH in late persisters (pH 6.9 ± 0.1 in S20 vs. pH 7.5 ± 0.1 in Log, Fig. 3f), which reflected the accumulation of 3-ketoacids (BHB, p*K*_a_ = 4.7; AcAc, p*K*_a_ = 3.6)^36,37^. Furthermore, starved persisters remained more viable in acidic growth conditions (pH 5.0) than log-growing and R6 cells (Supplementary Fig. 5i, k), suggesting that ketoacidosis may be an adaptive mechanism for mycobacterial persistence. Together, our findings indicate that mycobacterial persisters undergo a metabolic shift towards fatty acid β-oxidation, accumulate resulting ketone bodies, and adapt to ketoacidosis during nutrient starvation.

To further investigate the metabolic consequences of nutrient deprivation in BCG, we performed time-course metabolomics to define shifts in metabolite pools during starvation and resuscitation. Here we used chromatography-coupled mass spectrometry to quantify 64 metabolites at five points on the starvation time course (Log, S4, S10, S20, and R6; Supplementary Data 3 and Supplementary Fig. 6). It is important to note that measurement of steady-state levels of metabolites does not provide information about the dynamics or flux through pathways and must be considered holistically with the proteomics and phenotypic profiling data. Hierarchical clustering of the metabolomics data (Supplementary Fig. 6c) paralleled the phenotypic profiling (Fig. 3c, d) and highlighted significant shifts in metabolites associated with central carbon metabolism. For example, by S10, we observed increased levels of glyoxylate (twofold), malic acid (1.4-fold) and phosphoenolpyruvic acid (PEP, 1.4-fold) (Supplementary Data 3), which suggested increased metabolic flux through the glyoxylate shunt, agreeing with previous observations that glyoxylate shunting is important for carbon assimilation and mycobacterial survival during fatty acid metabolism^38^. However, it has been shown that glyoxylate is a toxic metabolic intermediate that can interact with proteins and DNA within the cell, as well interact with other metabolites such as oxaloacetate and pyruvate, resulting in products that can reduce levels of the corresponding metabolites and/or inhibit enzymes of the TCA cycle^9^. Consistent with these observations, by S10, we observed decreased pyruvate (52%) and markedly increased levels of the cataplerotic pyruvate-related intermediates alanine (540%), glycine (249%), tryptophan (234%) and threonine (40%) (Supplementary Data 3, Supplementary Fig. 7). In addition, by S20, we observed a 54-fold decrease in fructose-6-phosphate, a 31-fold decrease in glucose-6-phosphate, a tenfold decrease in succinate, a threefold decrease in citrate, and a 3.5-fold decrease in isocitrate levels relative to Log (Supplementary Data 3 and Supplementary Fig. 7). These decreases suggested a concurrent drop in the steady-state activity in the pentose phosphate pathway (PPP) and TCA cycle during late starvation, which is supported by the proteomics and metabolomics data shown in Supplementary Fig. 7.

Trends in other detected metabolites are consistent with the observed shift away from glycolysis and toward fatty acid oxidation. For example, cyclic AMP (cAMP) dramatically dropped below the limit of detection during starvation (Supplementary Data 3). Previous studies have revealed a close positive link between glucose metabolism and cAMP levels in mycobacteria^39^, and others have demonstrated that cAMP-dependent protein lysine acetylation regulates fatty acid and propionate metabolism in mycobacteria^40^. This finding parallels the observed importance of acetylation in the regulation of mitochondrial metabolic enzymes—including those involved in fatty acid β-oxidation—in eukaryotes^41^. For other metabolic pathways, there was an ironic increase in nearly all metabolic enzymes to some extent during starvation, while hypoxia causes more variable changes in enzyme levels (Supplementary Fig. 7). This behavior contrasts sharply with the levels of associated metabolites. For example, glycolysis and Kreb’s cycle metabolites decrease during starvation and increase during hypoxia. Similar behavior was observed for metabolites and enzymes in lactate oxidation, pentose phosphate metabolism, glutamate decarboxylation, and propionyl-CoA metabolism (including 2-methylcitrate cycle) (Supplementary Fig. 7). Interestingly, one of the only enzymes to decrease during starvation was isocitrate lyase Type I (Icl) (Supplementary Fig. 7), one of two isocitrate lyases along with Icl2 or AceA, which increased during starvation (Supplementary Fig. 7). Icl1 has no known function but both Icl1 and Icl2 are required for growth of *M. tuberculosis*^42^.

### A model for the management of starvation-induced ketosis in mycobacteria

While β-oxidation of fatty acids has previously been demonstrated in stressed mycobacteria^15^, the role of ketone bodies in mycobacterial persistence has not been characterized. We set out to identify a ketone body metabolic pathway in starved BCG, combining time-resolved, whole-genome RNA-seq (Supplementary Data 2) with quantitative proteomics (Supplementary Data 1) to define global responses to starvation. From RNA-seq, our analyses revealed an overall reduction in transcriptional activity during starvation (Supplementary Figs. 1e and 2a) and identified 511 upregulated transcripts (fold change ≥1.5 and *P* ≤ 0.05), as well as 680 downregulated transcripts (fold change ≤0.66 and *P* ≤ 0.05) (Supplementary Data 2). Using DAVID functional annotation and classification^43^, with cross-validation of pathway enrichments using KEGG Pathway Mapper^41^, we found the transcriptional response to starvation to be characterized by altered carbon and nitrogen metabolism, substrate translocation across membranes, environmental and intracellular signal transduction, and increased DNA repair, as well as RNA, protein, lipid and xenobiotic degradation (Supplementary Fig. 2c). The observation of pathway enrichment for transcripts for ketone body synthesis and degradation (Supplementary Fig. 2c) coincides with our biochemical observations in starved BCG (Fig. 3e–g). Likewise, comparison of the most significantly upregulated (>1.3-fold) proteins in S20 and R6 indicates marked differences in carbon metabolism, lipid catabolism, antigen secretion, translational regulation, divalent cation transport and heavy metal homeostasis (Supplementary Fig. 3).

**Fig. 4:**
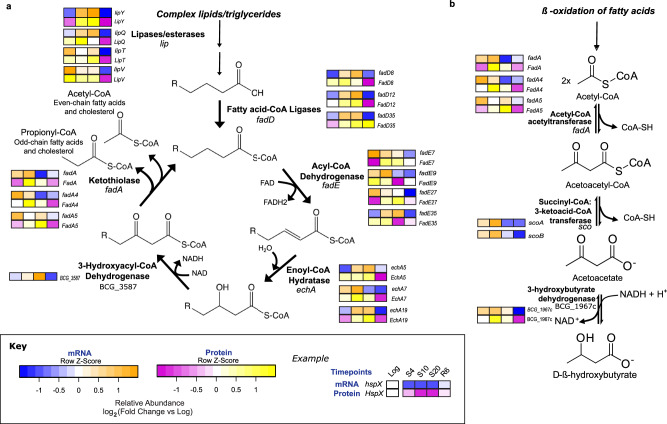
A proposed metabolic pathway that links fatty acid β-oxidation to ketogenesis in starvation-induced NRP mycobacteria. **a** Heatmaps show that starvation causes a coordinated transcriptional and translational upregulation of enzymes involved in fatty acid β-oxidation, with both transcription and translation downregulated upon resuscitation. The heatmap key is described below. This behavior underscores the importance of fatty acid metabolism for starvation-induced NRP in BCG. Upregulated *lipQ, lipT, lipV*, and *lipY* release fatty acids that are converted to fatty acid-CoA by fatty acid ligases (FadD; *fadD8, fadD12, fadD35*) as the entry point for β-oxidation. Subsequently, fatty acid oxidation is mediated by *fadE7, fadE9, fadE27,* and *fadE35*, 3-hydroxyl oxidation by *BCG_3587*, and hydration by *echA5, echA7,* and *echA19*, with the terminal thiolysis performed by *fadA*, *fadA4*, and *fadA5*. **b** Acetyl-CoA from fatty acid β-oxidation is then facilitates the starvation-induced increase in b-hydroybutryte (BHB; Fig. 3e). Starvation-upregulated ketothiolases (*fadA*, *fadA4*, *fadA5*) serve as acetyl-coA acetyltransferases to condense 2 acetyl-CoA molecules to acetoacetyl-CoA, which is converted to acetoacetate by upregulated succinyl-CoA:3-ketoacid-CoA transferase (*scoA, scoB*) and to BHB by upregulated 3-hydroxybutyrate dehydrogenase (*BCG_1967c*). Proteins levels for BCG_3587, ScoA, and ScoB were not quantified. Key: Relative transcript and proteins abundances are visualized after counts are normalized to their levels at Log (hence not visualized, crossed boxes) and log_2_ transformed. Heatmaps are used to represent mRNA (blue–orange scale) and protein (purple–yellow scale) levels at various time points (S4, S10, S20, and R6) in order with colors assigned based on their Z-scores across the time points. With yellow (protein) or orange (mRNA) for positive Z-scores and purple (protein) and blue (mRNA) for negative Z-scores. As an illustration, results for the chaperone HspX are shown. See Supplementary Data 4 for the source data used in these graphs.

Upregulation of starvation-induced ketone body synthesis and degradation was also apparent at the protein level (Supplementary Data 2). Though there is generally poor overall correlation between mRNA transcript and protein levels across all time points (explained variance in protein expression based on mRNA levels 0.18–0.54%; Supplementary Fig. 8), we found coordinated regulation of transcripts and proteins for fatty acid β-oxidation and BHB synthesis (Fig. 4a), with both mRNA and proteins upregulated in two or more starvation time points (i.e., S4, S10, S20) and then downregulated at R6 (Supplementary Fig. 2B).

The combined results of our biochemical, RNA-seq, and proteomic analyses, along with previous studies of stressed mycobacteria, suggest a novel pathway for the detoxification of ketone bodies in mycobacteria. Ketone body metabolism in mycobacteria has neither been characterized nor annotated in public databases, so we first attempted to identify detoxification pathways known from other prokaryotes but ruled out the occurrence of previously identified ketolytic pathways in our analysis through the process of elimination. For instance, some organisms are known to manage AcAc by converting acetoacetyl-CoA (AcAc-CoA) to 3-hydroxy-3-methylglutaryl-CoA through the mevalonate pathway, or by breakdown of AcAc-CoA to acetyl-CoA (Ac-CoA) by class II thiolases (FadA-A6). However, mycobacteria do not use the mevalonate pathway^44^, and thiolase II involvement could be counterproductive in this mechanism, as these bifunctional enzymes also mediate Claisen condensation of Ac-CoA to AcAc-CoA^45^. Accordingly, our proteomic analysis suggests that starved persisters increase flux toward AcAc, with concerted upregulation of lipases (LipQ, LipT, LipV, LipY), medium- and short-chain fatty acid-CoA ligases (FadD1, FadD8, FadD12 and FadD35), acyl-CoA dehydrogenases (FadE2, FadE7, FadE9, FadE27 and FadE35), keto acyl-CoA thiolases (BCG_3587) and thiolase II (FadA2, FadA4-5) at both the mRNA and protein levels in a stress- and time-dependent manner that is consistent with the conversion of Ac-CoA to AcAc-CoA (Fig. 4a, b). Further action by succinyl-CoA:3-ketoacid-CoA transferase (ScoA, ScoB) and 3-hydroxybutyrate dehydrogenase (BCG_1967c) can convert AcAc-CoA to AcAc and BHB— which was observed to increase during starvation (Figs. 3e and 4b).

### Ketogenesis: an Achilles’ heel for starvation-induced NRP mycobacteria

**Fig. 5:**
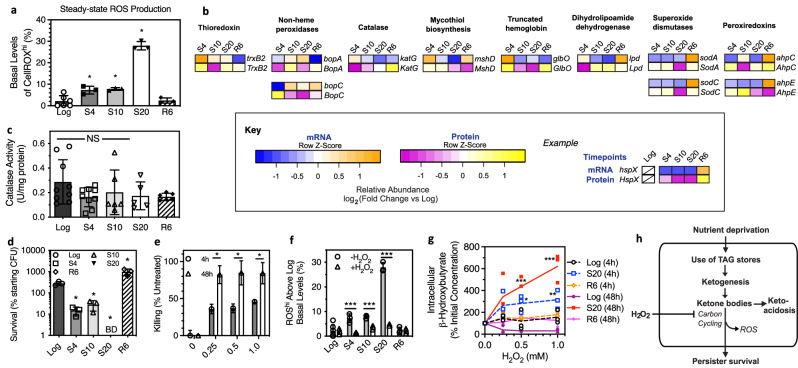
Dysregulation of ROS defense genes presents a vulnerability to H_2_O_2_ in ketogenetic persisters. **a** Starved BCG shows elevated levels of superoxide as detected by CellRox dye. Data represent the percentage of cells with elevated steady-state ROS production (CellROX^hi^) at different starvation time points. *N* ≥ 3; *P < 0.05; one-way ANOVA with Dunnett's test vs Log. **b** Heatmaps depicting transcriptional profiles and protein expressions for putative antioxidant response genes, showing a general lack of observable coordination between transcription and translation for these genes during starvation. **c** Catalase activity in Log, S4, S10, S20, and R6 cultures (*n* ≥ 6; *P > 0.05; one-way ANOVA with Dunnett's test vs Log). **d** Survival of BCG after 48 h of exposure to 0.5 mM H_2_O_2_. BD, below detection (1000 CFU). *N* = 6; *P < 0.05; one-way ANOVA with Bonferroni post test. **e** Killing of S20 BCG when expos**e**d to H_2_O_2_. S20 cultures (25 mL of ~10^8^ CFU/mL) were treated in 50 mL conical tubes at indicated H_2_O_2_ concentrations. *N* = 3; *P < 0.05, unpaired two-tailed *t* test with Welch’s correction. **f** Percentage of cells with elevated steady-state ROS levels (CellROXhi) following 4 h of mock or 0.5 mM H_2_O_2_ exposure. *N* = 8; *P < 0.05; unpaired, two-tailed *t* test of treatment groups with Welch’s correction. **g** Intracellular β-hydroxybutyrate levels in Log, S20, and R6 cells after 4 and 48 h of H_2_O_2_ exposure. *N* ≥ 3; *P < 0.05; two-way ANOVA with Bonferroni post tests comparing cell state and H_2_O_2_ dose. **h** Model of starvation-induced ketosis depicting conserved vulnerability of starved persisters to H_2_O_2_ blocking ketone body metabolism and thus carbon cycling for NRP survival. See Supplementary Data 4 for source data used in these graphs.

Ketosis has been linked to oxidative stress^46^ and we observed substantially elevated basal levels of superoxide in starved persisters (Fig. 5a) using CellRox Green, which has been established to be selective for superoxide in bacteria^47^. Intriguingly, despite highly elevated superoxide during starvation, we did not detect increased transcriptional induction of oxidative stress defense genes, apart from thioredoxins (Supplementary Fig. 9). Nor did we detect significant overexpression of these genes by proteomics (Fig. 5b), despite measuring HemB and HemC as two of the most upregulated proteins during starvation—both enzymes convert porphobilinogen to porphyrin to produce high levels of H_2_O_2_ (Supplementary Fig. 3a)^48^. As with our RNA-seq results, PCA of our proteomic results identified thioredoxin reductase (TrxB2) as the strongest positive covariant of starvation involved in ROS detoxification. Other putative ROS response proteins (e.g., BpoA, BpoC, KatG, GlbO, SodA, SodC, AhpC, AhpE) either remained relatively unchanged or were downregulated at some or all starvation time points measured. This suggests that the functional expression of genes involved in mycobacterial redox and ROS homeostasis is dysregulated during nutrient deprivation (Figs. 2b and 5b). For example, dihydrolipoyl dehydrogenase (Lpd) is an essential component of the β-ketoacid dehydrogenase complexes that—together with AhpC, AhpD, and DlaT—constitutes an NADH-dependent peroxidase active against hydrogen and alkyl peroxides, and also serves as a peroxynitrite reductase, protecting the bacterium against reactive nitrogen intermediates and oxidative stress generated by the host immune system^8^. As Lpd levels are increased in BCG during starvation at S10 and S20 and the MTB homolog of *lpd* (Rv0462) is essential for pathogenesis, this enzyme could enable ketogenic mycobacteria to cope with heightened ROS generation during starvation. However, PCA identified AhpC, AhpD, and DlaT, the other proteins in the β-ketoacid dehydrogenase complex, as strong transcriptomic contravariants of the starvation response, providing further evidence that starved BCG fails to genetically respond to increased intracellular ROS production.

In contrast to the proteomic response of starved mycobacteria, cell wall anabolic enzymes strongly covary with the overall proteomic response of hypoxic mycobacteria (Fig. 2b and Supplementary Fig. 3b). This agrees with previous reports that WhiB3 (in concert with DevS/R/T) modulates macrophage responses by regulating virulence lipid anabolism during redox stress, and that TAG formation serves as a convenient sink for the toxic reducing equivalents of NADH that accumulate due to impaired oxidative phosphorylation^49^. Collectively, these findings add to our understanding of how mycobacteria use complex and divergent metabolic strategies to survive and adapt to their equally complicated environments.

It was unexpected that the canonical ROS management enzymes (e.g., SOD, catalase) were not upregulated by the elevated superoxide in starved BCG. Indeed, KatG, SodA, and SodC protein levels were unchanged or slightly lower in late starvation at the peak of superoxide production (Fig. 5b), nor did we detect changes in catalase activity in these bacilli (Fig. 5c). Interestingly, SodC is a component of the mycobacterial respiratory supercomplex CIII_2_CIV_2_SOD_2_^50^, which raises the question of a link between ketone bodies and mitigation of the greatly increased superoxide production in starved BCG. Ketone bodies have indeed been demonstrated to have antioxidant properties presumably by increasing NADH oxidation (i.e., increasing the NAD + /NADH ratio) and increasing the efficiency of electron transport, thus reducing superoxide production^51^. However, the time-dependent but reversible increase in intracellular superoxide coinciding with BHB accumulation suggests otherwise (Figs. 5a and 3e). On the contrary, our transcriptomics, proteomics, and metabolomics data suggests that, unlike hypoxia-induced NRP^52^, starvation-induced NRP bacilli would be hypersensitive to the toxicity of superoxide such as that generated by hydrogen peroxide exposure, for example.

To test this hypothesis, we exposed starvation-induced NRP BCG to otherwise sublethal concentrations of H_2_O_2_. We observed that exposure to millimolar concentrations of H_2_O_2_ proved to be bactericidal to S10 and S20, but not to Log or R6 BCG in a time and dose-dependent manner (Fig. 5d, e). Hence, the mycobacterial adaptive mechanisms for surviving acidification (Fig. 3f), antibiotic exposure (Fig. 1f), and ROS generation (Fig. 5a) do not extend to the biocidal activities of H_2_O_2_. Interestingly, H_2_O_2_ treatment significantly lowered steady-state ROS levels (i.e., superoxide measured by CellRox Green^47^) in starved but not Log or R6 BCG (Fig. 5f). More importantly, H_2_O_2_ caused BHB to accumulate within 4 h of exposure, reaching over 600% of initial levels 48 h after H_2_O_2_ treatment in S20 but not Log or R6 cells (Fig. 5g). This effect on BHB levels paralleled H_2_O_2_-mediated killing, which was greater at 48 h than at 4 h following exposure (Fig. 5e.). These results directly link H_2_O_2_ cytotoxicity to the inhibition of ketone body metabolism for carbon cycling (Fig. 5h), which may relate to the well-established role of H_2_O_2_ as a cytochrome P450 (CYP) poison^53^.

## Discussion

The general concept of bacterial persistence arose over 70 years ago^54,55^ to describe the enhanced survival of subpopulations of bacteria subjected to antibiotic exposure. The tuberculosis community recognized a similar phenomenon in the form of a non-replicating persistence (NRP) that characterized the dormant-like state of mycobacteria deprived of oxygen or nutrients as models of the tuberculous granuloma, with slow or no growth of the bacteria and a reversible or phenotypic antibiotic tolerance^25,56^. While intensive study has been devoted to characterizing the mycobacterial response to both hypoxia and nutrient deprivation (e.g., references^3,16^), a central question had yet to be answered: how do two orthogonal stresses lead to comparable NRP phenotypes? Here we performed multiple systems-level analyses to interrogate the responses of the *M. tuberculosis* surrogate *M. bovis* BCG to the NRP-inducing stresses of hypoxia and starvation, emphasizing the latter given the deeper understanding of the molecular mechanisms governing the hypoxia responses. Considering recent investigations of mycobacterial metabolism^8^, our systems-level analysis of persister phenotype, biochemistry, and functional gene regulation revealed that these two very different stresses caused a coordinated shift away from glycolysis toward two distinct lipid metabolism-based pathways: lipid catabolism, fatty acid β-oxidation, and ketone body metabolism in starvation, versus cholesterol metabolism and anabolic triglyceride storage in hypoxia. That mycobacteria adapt to stress by pivoting on different lipid metabolic pathways is not surprising given the significant portion of their genomic coding capacity devoted to enzymes involved in lipogenesis and lipolysis^4,5^. Considering the mixture of environmental stresses that occur during mycobacterial infections, these results provide novel insights into the metabolic fine-tuning that is a hallmark of mycobacterial pathogenesis.

A major finding here involves starvation-induced ketosis. The accumulation of acidic ketone bodies such as BHB and AcAc is a hallmark of fatty acid β-oxidation in all organisms. The observation of ketone body accumulation and the resulting drop in intracellular pH during starvation-induced NRP points to ability of mycobacteria to manage the stress of low pH conditions. This may be a key to mycobacterial survival during acute infection if the bacilli are not immediately able to subvert phagolysosome biogenesis and acidification^6^. It may also be important to survival in chronic infection during which mycobacteria are sequestered within macrophages with nutrient deprivation triggering the NRP phenotype and causing ketone body accumulation and low intracellular pH. Lin et al. observed that Mtb exposed to presumably acidic lysosomal fractions from activated macrophages also switch to fatty acid β-oxidation, which reiterates the importance of this adaptive metabolism to mycobacterial survival^57^.

The ability of starved BCG to manage proton accumulation is supported by the significant upregulation of proton efflux pumps and glutamate decarboxylase (GadB) (Supplementary Table 2). Amino acid decarboxylation is a major mechanism of intracellular deacidification in bacteria, with the consumption of a proton when glutamate and arginine are decarboxylated^58^. Starved BCG showed significant upregulation of GadB while the enzyme was downregulated in hypoxic BCG (Supplementary Data 2 and Supplementary Fig. 4c). With regard to proton efflux pumps, the starvation-induced drop in intracellular pH in BCG is not large enough (pH 7.4 in PBS vs pH 6.9 intracellular; Δ0.5 pH units) to disrupt the proton motive force for ATP synthesis, which is essential in non-replicative mycobacteria^59^. Indeed, proton-driven ATP synthase (*atp* genes) is upregulated in starved BCG as compared to downregulation in response to hypoxia (Supplementary Data 2 and Supplementary Fig. 4), the latter also observed in other mycobacteria^59^. Proton efflux in starved BCG is likely managed in part by normal (i.e., nutrient-replete, aerobic) levels of the proton-pumping *aa*_3/_bc_1_ cytochrome oxidase complex (*qcr* genes) and proton-pumping type-I NADH dehydrogenase (NDH-1; *nuo* genes) (Supplementary Data 2 and Supplementary Fig. 4b). That succinate dehydrogenase (*sdh*)-driven menaquinone reduction drives the proton-pumping *aa*_3/_bc_1_ cytochrome oxidase complex is supported by the starvation-induced upregulation of Sdh levels (Supplementary Data 2 and Supplementary Fig. 4b), an eightfold reduction in succinate (Supplementary Data 3 and Supplementary Fig. 7), and upregulation of the menaquinone synthesis pathway (*menBEG*; Supplementary Data 2 and Supplementary Fig. 4b). In contrast, hypoxia upregulates the alternative *cd* cytochrome oxidase complex, which is a weak proton pump (i.e., low H^+^/e^−^ ratio) but has higher oxygen affinity than *aa*_3/_bc_1_^60^, and significantly downregulates Sdh and the proton-pumping *aa*_3_/bc_1_ cytochrome pathway (Supplementary Data 2 and Supplementary Fig. 4b), the latter consistent with a previous report^61^. The proton management features of starvation-induced persistence in BCG may explain the enhanced survival of persistent mycobacteria in acidic media, mimicking the intracellular environment of macrophages^37,62^ (Supplementary Fig. 5c–e).

In addition to mycobacterial acid management, the observed shift to ketosis in nutrient-deprived BCG may have implications for mycobacterial fitness during human infection. There is a well-established switch to lipid metabolism and ketogenesis that occurs during calorie-restricted, low-carbohydrate diets and in diabetes in humans, with greatly elevated blood levels of BHB, AcAc, and other ketone bodies^63,64^. Several pieces of evidence point to a ketosis-based survival advantage for mycobacteria in NRP. First, phenotypic profiling shows the survival of starved BCG with BHB and AcAc as the sole nutrients (Fig. 3c, d). While neutral ketone bodies such as acetone will freely diffuse across mycobacterial cell walls, it is reasonable to expect that negatively charged ketone bodies such as BHB and AcAc will be actively transported into the cell from the local environment by one of the numerous nutrient-scavenging transport proteins highly upregulated in starved BCG (Supplementary Data 2), including phosphate-specific transporter lipoproteins (PstS1), cysteine transporter (CydD), glutamine transporter (BCG 0103/0104), glycerol-3-phosphate (UgpC/A), and ribonucleotide transporter (BCG 0704). Second, starved BCG are primed for ketone body metabolism by upregulation of numerous metabolic enzymes (Fig. 4 and Supplementary Data 2), with inhibition of BHB metabolism by H_2_O_2_ leading to toxic BHB accumulation (Fig. 5g).

Though highly speculative, the dependence of starved, slowly dividing, antibiotic-tolerant mycobacteria on ketosis suggests a possible link to the epidemiological observation that people with diabetes and malnutrition have an increased risk for TB infection^65,66^. Not only do ketone bodies represent a nutrient source for stressed mycobacteria, but BHB has been shown to inhibit the NRLP3 inflammasome^67^, demonstrating that ketone bodies can directly regulate innate immune function in humans and could contribute to the systemic immune suppression previously proposed to underlie the co-morbidity of human diabetes and TB^65,66^. Of importance in human diabetes is the increase in the ratio of BHB to AcAc from a normal 1:1 to as high as 10:1 during in acute diabetic ketoacidosis^64^, which raises the question of a similar shift in starved, slowly dividing, antibiotic-tolerant mycobacteria.

The starvation-induced ketosis is also strongly linked to elevated superoxide levels possibly derived from CYP metabolism or leaky electron transport. Bacterial pathogens, including mycobacteria, are known to use CYPs in a range of essential biosynthetic and degradative pathways^68,69^, the importance of these enzymes for NRP has not previously been demonstrated. While we do not know yet which CYPs would be responsible for metabolism of ketone bodies, if at all, three observations support a tentative role for CYPs in the metabolic pathways for ketone bodies during starvation-induced NRP: (1) elevated transcript levels for 11 of 20 CYPs measured and for 3 of 8 quantifiable CYP protein genes in starved BCG (Supplemental Data 1 and 2), (2) greatly increased superoxide levels during NRP (Fig. 5a), and (3) a decrease in superoxide levels and increase in BHB levels caused by exposure to sublethal doses of the H_2_O_2_—a known CYP poison^53^ (Fig. 5f, g). Given the criticality of managing the load of ketone bodies during starvation-induced NRP, the enzymes involved in ketone body metabolism (Fig. 4) such as 3-hydroxybutyrate dehydrogenase, acetoacetate decarboxylase, and possibly CYPs may represent targets for development of small molecule inhibitors as antibiotics active against NRP bacteria.

While iron deprivation, nutrient starvation, and hypoxia are considered distinct stresses when modeling adaptative responses in mycobacteria^70,71^, several observations point to a central role for iron metabolism and heme-containing CYPs in metabolic carbon cycling of both cholesterol and ketone bodies in hypoxic and starved persisters, respectively. Our observations of the susceptibility of starvation-induced persisters to killing by CYP-inactivating H_2_O_2_ and by azole-based, Fe-center inactivating CYP inhibitors^72^ (Supplementary Fig. 10) parallel the development of inhibitors of cholesterol-metabolizing CYPs 125 and 142 as antimycobacterial agents^73^. The link to iron metabolism arises from observations that loss of IdeR, the iron-sensing transcription repressor/activator, increases the sensitivity of *M. tuberculosis* to H_2_O_2,_ again consistent with a critical role for heme-containing CYPs in mycobacterial physiology. Indeed, we observed that nutrient deprivation caused an early increase in IdeR mRNA (S4), with elevated protein levels throughout the starvation period (Supplementary Data 1 and 2), and increased levels of MmpL4 and MmpL5—transporters for the mycobactin iron siderophore (Supplementary Fig. S4a). CYP-mediated metabolism could thus be part of the missing link between restricting iron availability, cholesterol utilization, and carbon cycling—an emerging pathway crucial to intracellular survival in macrophages.

The described mechanism for fatty acid metabolism and metabolite detoxification highlights a potential vulnerability of certain mycobacterial persisters during infection and accentuates the criticality of virulence-modifying lipogenesis for host immune subversion. For example, it has been previously shown that mycobacteria synthesize PDIMs and phenolic glycolipids (PGLs) to recruit and infect permissive macrophages, while evading microbicidal macrophages^7,74^. Our biochemical findings suggest that ketotic NRP mycobacteria may be viable only in permissive (low-H_2_O_2_-producing) macrophages. For instance, diabetic bone marrow-derived macrophages have been shown to have impaired H_2_O_2_ release^75^. The ability of mycobacteria to fine-tune lipid catabolism and cell wall restructuring is key to their pathogenic success.

Of note, the observed shift to ketosis in nutrient-deprived mycobacteria parallels the switch to lipid metabolism that occurs during caloric restriction, low-carbohydrate diets, and in diabetes in humans. Furthermore, the dependence of starved, slowly dividing, antibiotic-tolerant mycobacteria on ketosis may clue a link to the epidemiological observation that people with diabetes and malnutrition have an increased risk for TB infection^65,66^. Systemic immune suppression has previously been proposed to underlie the co-morbidity of human diabetes and TB, but the potentially important role that host-derived ketogenic factors play in supporting TB pathogenesis has yet to be explored mechanistically. Emerging molecular evidence suggests the link is real: recent studies show that BHB inhibits the NRLP3 inflammasome^67^, demonstrating that ketone bodies can directly regulate innate immune function in humans.

Our model for starvation-induced ketogenesis in mycobacteria warrants further investigation, as it provides new insights into the underlying metabolic transitions required for mycobacterial survival in the context of clinically relevant physiological stress. Our results point to an adaptive metabolic plasticity of mycobacteria, with the potential for proportional shifts among metabolic phenotypes based on the balance of the various stressors during infection and macrophage phagocytosis. Additional biochemical characterization and genetic screens for essentiality will help to validate and assign functions to poorly or un-annotated genes in the proposed metabolic pathway. Furthermore, future efforts could directly assess the importance of ketone metabolism for in vivo mycobacterial infection, establish a molecular basis for the epidemiological link between diabetes and TB infection, or exploit the observation that starved mycobacterial persisters remain vulnerable to exogenous ROS to develop new anti-TB therapies.

## Methods

### Bacterial strains and culture conditions

*Mycobacterium smegmatis* MC^2^155*, Mycobacterium bovis* BCG str. Pasteur 1173P2, and *Mycobacterium tuberculosis* CDC1551 (American Type Culture Collection) were grown in roller bottles (5 rpm and 37 °C) in Middlebrooke7H9 (BD) supplemented with 0.5% (w/v) albumin, 0.2% (w/v) glucose, 0.085% (w/v) NaCl, 0.2% (v/v) glycerol and 0.05% (v/v) Tween 80 as nutrient-replete media. Unless specified otherwise, nutrient-deprivation medium consisted of phosphate-buffered saline (PBS; 137 mM NaCl, 2.7 mM KCl, 10 mM Na_2_HPO_4_, 2 mM KH_2_PO_4_) with 0.05% v/v tyloxapol, a non-hydrolysable detergent, adjusted to pH 7.4. Where specified, Dulbecco’s PBS (DPBS, Sigma-Aldrich) containing 0.5 mM MgCl_2_, 0.9 mM CaCl_2_, 137 mM NaCl, 2.7 mM KCl, 8 mM Na_2_HPO_4_, 1.5 mM KH_2_PO_4_ was supplemented with 0.05% v/v tyloxapol and used as a nutrient-deprivation medium. For CFU determinations, serial dilution plating was used to quantify colony-forming units (CFU) on 7H11 agar for MTB, and 7H10 agar for both BCG and SMG, both supplemented with 10% (v/v) oleic acid-albumin-dextrose-catalase (OADC). The starvation-induced model for NRP was performed by pelleting exponentially growing cultures (OD_600_ of 0.4–0.8), washing cell pellets twice in 37 °C PBS, and resuspending cells in PBS-tyloxapol (0.05%) for subsequent incubation at 37 °C. At specified time points, starved cultures were resuscitated for up to 10 days by pelleting and re-suspension in nutrient-replete 7H9. Prolonged starvation cultures were routinely assessed for contamination by microscopy (Gram, Ziehl-Neelsen, or auramine-rhodamine staining) and streaking on blood agar. The hypoxia-induced model for NRP was performed as previously described^27^. Specific compositions of growth media are detailed in Supplementary Information—Methods.

### Azole susceptibility testing

Log, S20, and R6 cultures were treated at OD_600_ 0.1 with clotrimazole, econazole or miconazole at indicated concentrations in 96-well plates for 2 days. CFU were obtained from plating serial dilutions at the indicated time points post-treatment on 7H10 agar after 3–4 weeks of incubation at 37 °C.

### Biochemical plate assays

For catalase determination, pellets from 10 mL of culture at indicated time points were resuspended in 0.5 mL of 50 mM phosphate buffer plus 0.1 mM phenylmethylsulfonyl flouride PMSF and added to ~100 µL of 0.1 mm silica/zirconium beads in 2 mL screw-cap tubes with O-rings. Cells were bead-beaten with a Tissuelyser II (Qiagen) with pre-chilled chambers at ambient temperature for 10 min at 50 Hz. Lysate (supernatant) was collected after centrifuging at 12,000× *g* for 15 min at 4 °C to pellet the beads and cellular debris. Lysate was aliquoted and stored at −80 °C until use. Catalase activity in the lysate was measured using a catalase assay kit (Sigma-Aldrich) adapted for a 96-well plate. Protein content in the lysates was measured the same day as catalase using a BCA assay according to the manufacturer’s protocol (Pierce, Thermo Scientific). Catalase levels were normalized to the protein content of each lysate. ATP levels were quantified in cell lysates as mentioned above. ATP levels in lysates were compared with a standard curve of ATP (Promega) with the BacTiter Glo Assay (Promega) following the manufacturer’s instructions. ATP levels were normalized to CFU in the culture. Levels of BHB were quantified using a β-hydroxybutyrate (Ketone Body) Colorimetric Assay Kit (Cayman Chemical). Cell cultures treated under indicated conditions were pelleted at respective time points (Log, S4, S10, S20, R6), washed twice in PBS-tyloxapol (0.05%) and lysed by bead-beating in 100 mM Tris-HCl (pH 8.5) with 10% ethanol (v/v). BHB levels were measured directly after bead-beating and normalized to CFU in the culture.

### Triacylglycerol analysis

Thin-layer chromatography was used for semi-quantitative analysis of lipid content, as described in ref. ^76^. To extract lipids for triacylglyceride analysis, lyophilized and weighed cell pellets were lysed in methanol/petroleum ether (2:1) with 0.02% (w/v) sodium chloride with the volume proportional to dry weight. Soluble extracts were dried, dissolved in petroleum ether then spotted onto silica TLC plates with hexane/ethyl ether/acetic acid (45:5:1) as running buffer and iodine vapor as developing agent. Densitometry was performed using ImageJ (v1.49) (NIH), calculating pixel density based on the R_f_ of TAG standards.

### RNA extraction

To ensure complete lysis of mycobacteria, ~10^9^ pelleted cells were combined with 1 mL of TRIzol reagent (Life Technologies) and 100 µL of 0.1 mm silica/zirconium beads in a 2 mL screw-capped tube and shaken vigorously on a Qiagen Tissuelyser II (Qiagen) in pre-chilled chambers for 15 min at a frequency of 30 Hz. Lysates were chilled at −20 °C and a second beat-beating cycle was performed in the presence of 16% chloroform (v/v). Phase separation and RNA extraction was performed using the PureLink RNA Mini Kit (Life Technologies, Carlsbad, CA). A two-column separation strategy was used with on-column DNaseI digest: 35% ethanol (v/v) on the first column to enrich for long (>~150 nt) RNA species. 70% ethanol (v/v) was used on the second to trap small (~150 to ~20 nt) RNA species. Concentration was then quantified by UV spectroscopy at 260/280 nm. RNA integrity and composition was determined using the appropriate Bioanalyzer RNA chips (Agilent Technologies). Only samples with an RNA Integrity Number (RIN) of 8.0 or greater were used for subsequent RNA-Seq and qPCR experiments. Relative quantities of each non-coding RNA species were determined using RNA liquid chromatography as described elsehwere^77,78^ For RNA-seq, rRNA is depleted using the Ribo-Zero Magnetic Kit for Bacteria (Epicentre, Illumina) as per manufacturer’s instructions.

### Quantitative real-time PCR

DNA-free RNA was diluted to a concentration of 50 ng/µl and reverse-transcribed using the iScript cDNA synthesis kit (Bio-Rad) according to the manufacturer’s instructions. The reverse transcription program was run as follows: 25 °C for 5 min, 42 °C for 30 min and 85 °C for 5 min, followed by a cooling step at 4 °C. Two-step real-time qPCR was performed by using the Sso Advanced Universal SYBR Green Supermix (Bio-Rad). The list of primers and their respective sequences and melting temperatures can be found in Supplementary Table 6. The qPCR program was run as follows. In all, 95 °C for 30 s followed by 40 cycles of denaturation at 95 °C for 15 s and annealing/extension at 60 °C for 30 s. A melt curve analysis consisting of 0.5 °C increments from 65 to 95 °C was performed for all reactions to ascertain the specificity of the primers. Sample C_T_ values were normalized against endogenous *sigJ* expression and analyzed using the comparative C_T_ method.

### BCG starvation proteomics: isobaric labeling and peptide fractionation

As the first step in the quantitative analysis of the starved BCG proteome, proteins were extracted^27^ from biological triplicate cultures of BCG harvested during logarithmic growth in 7H9 and from cultures washed and resuspended in PBS for 4, 10, and 20 days (S4, S10, S20, respectively) and then resuspended in 7H9 medium for 6 days (S-R6). The extracted proteins were then precipitated, quantified, and processed as previously described for BCG hypoxia proteomics^27^. Aliquots of trypsin-digested protein (from 50 µg of total protein) were labeled with TMT 6-plex reagents (Thermo Scientific Tandem Mass Tag Reagents) according to the manufacturer’s instructions. Aliquots (5 µL) of the labeled peptides mixture were removed from each biological replicate and combined equi-volumetrically to reconstitute a full label set, which was analyzed on a Thermo Scientific EASY-nLC 1200 interfaced to a Thermo Scientific Q Exactive Hybrid Quadrupole-Orbitrap MS. Median total ion intensities for each label were calculated and used to normalize volumetric mixing of the remaining respective labeled samples, so as to avoid signal suppression or bias from any one label. The 6-plex mixture was then desalted with C18 SpinTips (Protea), dried by vacuum centrifugation, and reconstituted in Agilent IPD buffer without glycerol (7 M urea, 2 M thiourea, 1% DTT, 1% IPG buffer pH 3-10). Isoelectric focusing was performed from pH 3 to 10 over 24 wells on an Agilent 3100 OFFGEL fractionator according to the manufacturer’s protocol (OG24PE00). Each of the 24 fractions was collected, dried by vacuum centrifuge, resuspended in 0.1% formic acid in water, and analyzed by nano-LC-MS/MS.

### BCG starvation proteomics: Nano-LC-MS/MS analysis of the BCG proteome

The TMT-labeled starvation time-course samples were analyzed on an Agilent 1200 nano-LC-Chip/MS interfaced to an Agilent 6550 iFunnel Q-TOF LC-MS. The LC system consisted of a capillary pump for sample loading, a nanoflow pump, and a thermostated microwell-plate autosampler. The HPLC-Chip configuration consisted of a 160-nL enrichment column and a 150 mm × 75 µm analytical column (G4340-62001 Zorbax 300SB-C18). The following mass spectrometry grade mobile phases (Burdick & Jackson) were used: 0.1% formic acid in water (solvent A), and 0.1% formic acid in acetonitrile (solvent B). A 130-min linear gradient LC separation was used with 10 min for column wash and equilibration between runs. Samples (1–2 µL injections) were loaded onto the enrichment column at 3% (*v*/*v*) B at flow rates of 3 µL min^−1^. The analytical gradient of solvent B was performed at a constant flow rate of 0.3 µL min^−1^ using the following solvent transitions on the nanoflow pump: 0–1 min, held at 1% (*v*/*v*); 1–10 min, 1–15%; 10–101 min, 15–35%; 101–121 min, 35–75%; 121–123 min, 75–98%; 123–126 min, held at 98%; 126–127 min, 98–1%; 127–130 min, held at 1%. LC-Q-TOF was operated at high sensitivity (4 GHz) in positive ion mode with the following source conditions: gas temperature 325°C, drying gas 13 L min^-1^, fragmentor 360 V. Capillary voltage was manually adjusted between 1800 and 2150 V to achieve a steady nanospray. Data were acquired from 300 to 1700 *m/z* with an acquisition rate of 6 spectra s^−1^ in MS mode, and from 50 to 1700 *m/z* with an acquisition rate of 3 spectra s^−1^ in MS/MS mode. A peptide isotope model (charge state 2 + ) was used to detect a maximum 20 precursors per cycle at a minimum threshold of 25,000 counts/spectra at a narrow isolation window (~1.3 *m/z*). Sloped collision energy (C.E.) was used to maximize collision-induced dissociation of detected isobarically tagged peptides according to the following rules: charge state 2 + C.E. slope 4.2, offset 3.5; charge states ≥3 + C.E. slope 4.2, offset 4.

LC-MS data was extracted and evaluated for quality using the MFE algorithm in MassHunter Qualitative Analysis software (v B06.00). Test injections (three to four) from each fraction of the first technical replicate were used to optimize injection volumes for the second and third biological replicates with the aim to maximize the number of extracted molecules with peptide-like features. For each fraction, the MFE list of molecular ions was exported and used to exclude the spectral acquisition of these ions in subsequent technical replicates. Each of the 24 fractions from biological triplicates were injected in technical duplicate—spectra generated from technical replicates 1 were acquired without use of an exclusion list, whereas spectra generated from technical replicate 2 were acquired with the exclusion list. Data from MassHunter Qualitative Analysis was exported to Mass Profiler Professional (v B03.00) for analysis of technical reproducibility. This process was repeated for all three biological replicates. Mass spectra were processed using Spectrum Mill (Agilent, v B06.00) and Scaffold Q+ (v Scaffold_4.8.8), and quantified protein associations were manually analyzed in Excel. Manually analyzing data pre-filtered at a 95% confidence interval (2 peptide minimum per protein ID) yielded 1217 highly quantifiable proteins for the starvation proteomics experiment. Similarly, 965 highly quantifiable proteins were identified in all time points of our published BCG hypoxia iTRAQ proteomics studies^27^. The hypoxia proteomics data are available from the CHORUS mass spectrometric data repository at https://chorusproject.org/; Project ID 1107. Starvation proteomic data are available from the at Chorus Project (https://chorusproject.org/pages/index.html).

### Antibiotic, formaldehyde, and hydrogen peroxide susceptibility testing

Log, S4, S10, S20, and R6 cultures were treated at OD_600_ 0.1 with streptomycin, rifampicin, ethambutol, isoniazid, methanol-free formaldehyde or H_2_O_2_ at indicated concentrations in 96-well plates for up to 5 days. CFU counts were obtained from plates of serial dilutions at the indicated post-treatment time points on 7H10 agar following 3–4 weeks of incubation at 37 °C (for MTB and BCG), and 2–3 days (for SMG). Similar results for starvation-induced antibiotic resistance were reported previously in ref. ^47^.

### Flow cytometry of cellular physiology

For staining, 20 μl of the sample was added to a well of a v-bottom 96-well plate containing 180 μL of 500 nM CellROX Green (Life Technologies) in PBS then incubated at 37 °C for 30 min. Following incubation, bacteria were stained for DNA and fixed by the addition of 50 μL of 4% PFA containing DAPI (1 μg/mL) for 10 min. Samples were analyzed on an LSRII HTS flow cytometer (BD Biosciences) within an hour of staining with >50,000 events collected per sample when possible. Data were analyzed using FlowJo software (TreeStar Inc). ROS^hi^ bacteria were identified based on a sequential gating scheme as shown in Supplementary Fig. 2f for: (1) size (FSC and SSC), (2) DNA content (DAPI), (3) ROS detection dye uptake (CellROX signal above unstained), and (4) ROS^hi^ above basal (CellROX signal above untreated). CFDA^hi^ cells were identified via a similar staining protocol (1 ng/mL CFDA, Life Technologies) and gating scheme (Supplementary Fig. 2b).

For pH determination, cells were pelleted, washed once with 100 mM phosphate buffer, pH 7.0, and resuspended at ~2 × 10^6^ cells/ml in carboxy SNARF 5F-AM acetate (SNARF, 10 μM, Life Technologies) in 100 mM phosphate buffer, pH 7.0 or buffer only for 30 min. Additional aliquots of cells were used for a calibration curve in which SNARF plus nigericin (10 μM, Life Technologies) in 100 mM phosphate buffers at pH 5.5–8.0 was added to cells. Every condition and flow analysis had a separate calibration curve. DAPI (1 μg/mL) was added to all cells for at least 10 min prior to flow cytometry. SNARF was excited at 561 nm with emission at 585/15 and 610/20. Bacteria were identified by size and DNA content, as described above. pH was calculated according to the manufacturer’s instructions using the ratios of median fluorescent intensity (MFI) of SNARF at the two wavelengths. Photon multiplier tube (PMT) voltage settings for the LSRII were SSC (250), FSC (400), DAPI ex 355/em 450/50 (550), CellROX and CFDA excitation 488 nm and emission 525/50 (400), SNARF 585/15 (550), and SNARF 610/20 (550). A minimum of 50,000 events were collected for analysis from each sample.

### Metabolic phenotype assays

To adapt the standard GENIII microplate protocol (Biolog) to provide relative quantifications of metabolite usage, we first optimized for the survivability and tetrazolium reduction of BCG in either IF-C or IF-B media (Biolog) in the presence or absence of 7H9 (0–20% v/v) at various cell densities (from starting OD_600_ of 0.01–0.2) and for periods from 1 to 7 days at 37 °C based on reported protocols^30,31^. Full UV-VIS spectroscopic scans from 300 to 700 nm were performed on a Synergy 4 Multi-Mode Microplate Reader at 25 °C every 8 h to track the conversion of tetrazolium to formazan and changes in culture turbidity. Data was loaded unto Origin (v8.5) (OriginLab) for background subtraction, normalization, deconvolution (between tetrazolium reduction and optical density) and peak integration. Design of Experiments (DOE) was computed on Unscrambler X (Camco) and a starting OD_600_ of 0.05 with 5 days of incubation was optimal while A_565_ and OD_660_ were determined to minimize spillover from between tetrazolium reduction to optical density. Test plates were then performed to ascertain the sensitivity (limit of detection, LOD) for A_565_ and OD_660_ based on their initial values prior to incubation, by spiking in known volumes of reduced dye or varying the cell density. Metabolites that induced had A_565_ readings below LOD were arbitrarily assigned a null value, while those that had OD_660_ at or below the initial values, but A_565_ readings above LOD were considered to induce abiotic reduction of the dye. For actual sample plates, the contents of each well were plated onto 7H10 agar after incubation and A_565_ and OD_660_ reads to determine culture survivability. We noted that several wells that assayed for chemical sensitivity gave A_565_ readings below that of the negative control and a design decision was made to exclude these data from subsequent statistical modeling.

### Metabolomic profiling

Metabolite extraction followed the published reports^32,33^. Briefly, cell cultures were harvested at given time points, rapidly quenched and spun down. Cell pellets were resuspended in acetonitrile:methanol:water (2:2:1 *v*/*v*/*v*) and lysed mechanically with 0.1-mm silica beads by using Qiagen TissuelyserII. The lysates were collected and evaporated to dryness in a vacuum evaporator. The dry extracts were redissolved in 100 µL of 50:50 water/methanol for liquid chromatography–mass spectrometry (LC-MS) analysis.

Untargeted metabolomics were performed as previously described^34^. The redissolved dry extracts were analyzed using Agilent 1290 ultra-high-pressure liquid chromatography system equipped with a 6520 quadrupole time-of-flight (Q-TOF) mass detector managed by a MassHunter workstation. The column used for the separation was an Agilent rapid resolution HT Zorbax SB-C18 (2.1 × 100 mm, 1.8 mm; Agilent Technologies, Santa Clara, CA, USA). The oven temperature was set at 45 °C. The gradient elution involved a mobile phase consisting of (A) 0.1% formic acid in water and (B) 0.1% formic acid in methanol. The initial condition was set at 5% B. A 7 min linear gradient to 70% B was applied, followed by a 12 min gradient to 100% B which was held for 3 min, then returned to starting conditions over 0.1 min. The flow rate was set at 0.4 ml/min, and 5 mL of samples was injected. The electrospray ionization mass spectra were acquired in positive ion mode. Mass data were collected between *m/z* 100 and 1000 at a rate of two scans per second. The ion spray voltage was set at 4000 V, and the heated capillary temperature was maintained at 350 °C. The drying gas and nebulizer nitrogen gas flow rates were 12.0 L/min and 50 psi, respectively. Two reference masses were continuously infused to the system to allow constant mass correction during the run: *m/z* 121.0509 (C_5_H_4_N_4_) and *m/z* 922.0098 (C_18_H_18_O_6_N_3_P_3_F_24_). Raw spectrometric data were analyzed by MassHunter Qualitative Analysis software (Agilent Technologies, USA) and the molecular features characterized by retention time (RT), chromatographic peak intensity and accurate mass, were obtained by using the Molecular Feature Extractor algorithm. The features were then analyzed by MassHunter Mass Profiler Professional software (Agilent Technologies, USA). Only features with an intensity ≥20,000 counts (approximately three times the limit of detection of our LC-MS instrument) and found in at least 80% of the samples at the same sampling time point were kept for further processing. A tolerance window of 0.15 min and 2 mDa was used for alignment of RT and *m/z* values. Residual protein content was determined to normalize samples to cell biomass (BCA protein assay kit; Thermo Scientific).

The targeted LC-MS/MS analysis followed a published report with some modifications^32,35^. Briefly, LC-MS analysis was performed with Agilent 1290 ultra-high-pressure liquid chromatography system (Waldbronn, Germany) coupled to an electrospray ionization with iFunnel Technology on a triple quadrupole mass spectrometer. Chromatographic separation was achieved by using Atlantis HILIC column (2.1 × 100 mm, 1.7 µm; Waters, Eschbornn, Germany) with mobile phases (A) 10 mM ammonium formate and 0.1% formic acid in water and (B) 0.1% formic acid in acetonitrile. The initial condition was set at 100% B for 2 min, followed by a linear gradient to 80% B over 11 min and then down to 40% B over 1 min which was held for 5 min. Then the gradient returned to starting conditions over 1 min. The column was kept at 45 °C and the flow rate was 0.4 mL/min. The column used for the separation of glycolysis intermediates was Phenomenex (Torrance, CA) Rezex^TM^ ROA-Organic Acid H+ (8%) column (2.1 × 100 mm, 3 µm) and the compounds were eluted at 40 °C with an isocratic flow rate of 0.3 mL/min of 0.1% formic acid in water. The autosampler was cooled at 4 °C and an injection volume of 2 μL was used. Electrospray ionization was performed in both positive and negative electrospray ionization modes with the following source parameters: drying gas temperature 250 °C with a flow of 14 L/min, nebulizer gas pressure 40 psi, sheath gas temperature 400 °C with a flow of 11 L/min, nozzle voltage 500 V, and capillary voltage 4000 V and 3000 V for positive and negative mode respectively, and for positive and negative modes. Metabolites were measured and quantified in multiple reaction monitoring (MRM) mode and data acquisition and processing were performed using MassHunter software (Agilent Technologies, USA).

### RNA sequencing and transcriptome analysis

Coding sequences were enriched through rRNA and tRNA depletion and converted into template libraries using TruSeq RNA Sample Preparation v2 kit (Illumina) according to the manufacturer’s instructions. Pre-sequencing quality controls for fragment sizes, dsDNA concentrations, and loading molarities were performed. Samples were then pooled, loaded onto MiSeq Reagent Kit v2 cartridges (Illumina), and analyzed on a MiSeq System set at paired-end 151 bp reads. Data from technical replicates were pooled, and post-sequencing quality controls of transcriptome coverage, RNA purity, and RNA quality were determined (Supplementary Table 5). Sequencing reads were mapped using the BWA-MEM algorithm (http://bio-bwa.sourceforge.net) referenced against *Mycobacterium bovis* BCG Pasteur 1173P2, complete genome (GenBank: AM408590.1). Gene expression level was determined based on sequencing coverage of the gene boundaries defined. Appending the reads from independent technical duplicates of each of the three biological replicates, 1–2 million read pairs with a 3′-to-5′ ratio of 1.02 ± 0.03 for each sample, and >90% coverage of the transcriptome for all time points were obtained (Supplementary Table 5). Transcriptional profiling data were uploaded to the NCBI Gene Expression Omnibus (http://www.ncbi.nlm.nih.gov/geo/) as study GSE66883.

### Statistics and reproducibility

Comparisons between two samples were made using the appropriate two-tailed *t* tests after the equality of variances were tested using *F* tests. Comparisons between multiple samples were determined using one-way or two-way ANOVA when comparing 1 or 2 factors, respectively. Bonferroni’s or Dunnett’s Multiple or Control Comparison or Tukey’s HSD post hoc tests were used as stated in figure legends. Trends in antibiotic tolerances were determined using the Mann–Kendall trend test. Unless otherwise stated, all data are represented as arithmetic means ± SE.

Cluster analysis was performed using two-way hierarchical clustering with Euclidean distances and complete linkages. Exploratory data analysis was performed with PCA using the NIPALS method (100 iterations). Metabolic utilization predictors for cell state were determined using PLS-DA and tested on independent S30 and Log samples.

Differential RNA-seq expression analyses were carried out using DeSeq (http://genomebiology.com/2010/11/10/r106). The three replicates in each timepoint were compared against three control replicates as independent DeSeq statistical tests. The local fit option was used to estimate dispersions in order to reach convergence. Transcripts of interest were defined by at least 1.5-fold up- or downregulation and with multi-testing adjusted *P* values ≤ 0.05, and proteins of interest were defined by at least 1.3-fold up- or downregulation.

GSEA (Broad Institute) was performed for targets identified by RNA-seq by pair-wise comparisons of Log samples against S4, S10, S20, and R6 samples. Outliers were identified by Grubb’s tests and excluded, and 1000 permutations were performed. Genes associated with the ketone body-CYP Reactome were identified by RNA-seq based on a combination of significant gene expression change upon starvation (absolute fold change >1.5 at any one time point; *P* < 0.05), in addition to comprehensive functional annotation searches against DAVID (http://david.abcc.ncifcrf.gov/), Reactome (http://www.reactome.org/), TbDB (http://www.tbdb.org/), Tuberculist (http://tuberculist.epfl.ch/), BioCys (http://biocyc.org/) and KEGG databases. Genes whose functions and expressions agreed with previously reported phenotypes were defined as the high-confidence ketone body Reactome genes and used as a gene set. Expression data curated on NCBI Gene Expression Omnibus (http://www.ncbi.nlm.nih.gov/geo/) study GSE66883.

### Ethics and inclusion statement

The authors declare that the research reported here has included local researchers throughout the research process and is locally relevant to the public health concerns of Singapore and the USA, which was determined in collaboration with local partners. Roles and responsibilities were agreed among collaborators ahead of the research and revised as the research progressed in consultation with all authors, with no restrictions or prohibitions in the setting of the researchers. The research conducted here involved health and safety risks to researchers, including work with BSL2 microorganisms. All researchers were trained in the safe management and disposal of biohazardous materials in accordance with local rules and regulations.

### Supplementary information

Supplementary Information

Description of Additional Supplementary Materials

Supplementary Data 1

Supplementary Data 2

Supplementary Data 3

Supplementary Data 4

## Data Availability

All data are available on public databases or upon request to the authors. RNA sequencing data are available on the NCBI Gene Expression Omnibus (http://www.ncbi.nlm.nih.gov/geo/) as study GSE66883. Previously published hypoxia proteomics data are available from the CHORUS mass spectrometry data repository as Project ID 1107 (https://chorusproject.org/). Starvation proteomics data are available from the PRIDE database as ProteomeXchange accession PXD046804.
