# Supplementary material for: Facile metabolic reprogramming distinguishes mycobacterial adaptation to hypoxia and starvation: ketosis drives starvation-induced persistence in M. bovis BCG
Source: Commun Biol. 2024 Jul 16;7:866. doi: 10.1038/s42003-024-06562-2 (PMC11250799; doi:10.1038/s42003-024-06562-2)
Supplement: Supplementary file 1 — Supplementary Information [file 42003_2024_6562_MOESM1_ESM.pdf]

**Supplementary Information for**

# Facile metabolic reprogramming distinguishes mycobacterial adaptation to hypoxia and starvation: Ketosis drives starvation-induced persistence

Nick K. Davis, Yok Hian Chionh, Megan E. McBee, Fabian Hia, Duanduan Ma, Cui Liang, Mariam Lucila Sharaf, Weiling Maggie Cai, Watthanachai Jumpathong, Stuart S. Levine, Sylvie Alonso, Peter C. Dedon

**Contents:**

Supplementary Data

Supplementary Figures 1-7

Supplementary Table 1: Changes in forward and side scatter light during starvation

Supplementary Table 2: Changes in metabolism (tetrazolium reduction 485 nm) when BCG from log, S4, S10, S20, and S20R6 were transferred to single carbon source or chemical

Supplementary Table 3. Changes in growth (OD 660nm) when BCG from log, S4, S10, S20, and S20R6 were transferred to single carbon source or chemical

Supplementary Table 4. Variable Importance in Projection (VIP) scores and Kruskal-Wallis p-values from PLS-DA model of the metabolic phenotypes of Log, S4, S10, S20, and R6 cultures.

Supplementary Table 5. Replicate numbers, sequencing depth, and quality control parameters for RNA-seq of BCG transcriptome before, during, and after starvation.

Supplementary Table 6. Primers for qPCR

Supplementary Data 1. Proteomic analysis of starved BCG – see attached Excel spreadsheet

Supplementary Data 2. RNA-seq data for Starved BCG – see attached Excel spreadsheet

Supplementary Data 3. Metabolomic analysis of starved and hypoxic BCG – see attached Excel spreadsheet

Supplementary Data 4. Source data for all figures and supplementary figures – see attached Excel spreadsheet

Supplementary Discussion

Supplementary References

**Supplementary Data**

***Divalent cations support survival during the adaptive phase of starvation.*** Two forms of phosphate-buffered saline (PBS) are commonly used in research, PBS and Dulbecco's PBS (DPBS). In addition to starving BCG in PBS, we also evaluated use of DPBS as a carbon and nutrient deprivation medium. When starved in $Mg^{2+}$- and $Ca^{2+}$-containing DPBS, significantly more BCG survived compared to starvation in PBS (**Supplementary Fig. 1b**). We also observed this phenomenon in SMG, whereby >99% of the inoculum was recovered after starvation in DPBS compared with 30% recovery after starvation in PBS (**Supplementary Fig. 1c**). The SMG survival profile during nutrient deprivation in PBS agrees with prior characterizations performed by Stallings *et al.*[1]

***Cannibalism is not a major nutrient source for starved mycobacteria.*** We assessed starved cultures for the possibility that non-viable cells (~95% of initial CFU) served as a carbon source for viable starved BCG persisters in culture. After 20 days of starvation in PBS, cultures were split, pelleted at low speed (so as to pellet viable cells, while excluding "ghosts"), and either (1) washed and reconstituted to initial volume in fresh PBS, or (2) resuspended in its own (native) supernatant. We subsequently starved both cultures for an additional 20 days. We detected negligible differences in CFU recovery from cultures starved in fresh PBS against those reconstituted in native supernatant (**Supplementary Fig. 1d**). Ostensibly, cannibalism would reduce recovery from washed cultures, but this was not observed, suggesting that primarily metabolic quiescence or cellular autophagy underlies mycobacterial survival during extended starvation.

***Starved BCG persisters better tolerate acidic growth conditions.*** Our observation that the intracellular contents of BCG acidify during starvation compelled us to investigate whether starved bacilli could better survive growth in low-pH media. We used a GENIII MicroPlate screen for metabolic phenotype (see Results and **Fig. 3** in main text), to measure differential tolerance of acidic pH. While we observed no changes in cellular viability (recoverable CFU) during growth in pH 6.0 media, late-starved BCG (S10

and S20) were progressively more tolerant to pH 5.0 media than Log and R6 cells (**Supplementary Fig. 5i-k**). This differential acid tolerance was also observed by measuring optical densities and tetrazolium dye reduction, an indicator of cellular respiration (**Supplementary Fig. 5i-k**)[2,3].

***RNA-seq reveals global transcriptional reprogramming in starved persisters.*** Our rRNA and tRNA depletion strategy greatly enriched multi-gene operon cluster mRNAs for analysis by RNA sequencing (RNA-seq; see **Methods**). However, our preparatory approach did not remove the total content of non-coding RNA (ncRNA, **Supplementary Table 3**), providing a stoichiometric depiction of how ncRNA contributes to BCG's genetic response over the course of starvation. For instance, one of the most prominent and quantifiable ncRNA was the ribozyme *rnpB* (**Supplementary Fig. 3a; Supplementary Table 3**). Together with *rnpA,* these two proteins form the RNaseP complex, which catalyzes the removal of the 5'-leader sequence from pre-tRNAs in the presence of divalent cations ($Mg^{2+}$ or $Ca^{2+}$) to produce the mature tRNAs[4,5]. Continued *rnpB* transcription agrees with the observed maintenance of tRNAs especially during early starvation (**Supplementary Fig. 1e**) and challenges the notion of translational quiescence in persistent mycobacteria. Another prominent ncRNA observed across starvation time points was tmRNA, coded by *ssr* (**Supplementary Fig. 3a; Supplementary Table 3**). tmRNA is involved in trans-translation by releasing mRNA from stalled ribosomes, which facilitates translational fidelity[6]. The preservation of tmRNA during starvation further supports the active translation of functional proteins in mycobacterial persisters.

In bacteria, RNA synthesis is initiated by sigma factors. Specialized sigma factors, such as SigF, are induced under stress, bind their target promoters, and induce the transcription of stress response genes. Using Gene Set Enrichment Analysis (GSEA), we detected a significant enrichment of the SigF regulon at S10, despite peak *sigF* induction at S4 (**Supplementary Table 3**). A plausible explanation for this phenomenon may be the spikes in *rsbW-usfY* transcription at S4 (**Supplementary Fig. 2a; Supplementary Table 3**). *rsbW* codes for an anti-sigma factor protein that binds and negatively regulates

SigF activity[7]. This suggests a layer of regulation governing the dynamic transcription of stress response genes.

In addition to the SigF regulon, CYPs, and antioxidant defense genes (**Supplementary Fig. 2a-c; Supplementary Table 3**), PCA of RNA-Seq results also identified several genes involved with the TCA cycle or members of the Lsr2 regulon to co-vary with metabolic utilization phenotypes at various time points. These include *sucD*, *fum*, *acn*, *aceA* and *glcB* (TCA cycle), *sigF*, *canA*, *cyp12*8, *BCG_2905* (SigF regulon), and *moaD1*, *PPE16*, *PE9*, *celA2b* (Lsr2 regulon) (**Supplementary Fig. 2b; Supplementary Table 3**). GSEA further demonstrated that, relative to Log cultures, TCA cycle gene transcripts are significantly repressed by S4, but return to Log levels upon resuscitation (R6; **Supplementary Fig. 2c; Supplementary Table 3**). One notable exception to this generalizable trend is *aceA,* encoding isocitrate lyase, which is up-regulated at S4. In contrast, Lsr2 regulon gene transcripts are significantly induced at S20, but deplete by R6. Importantly, Lsr2 is a transcription repressor whose targets upregulate only upon its suppression; concomitantly, its targets are again repressed upon Lsr2 induction at R6 (**Supplementary Fig. 2b; Supplementary Table 3**). We also observed contributions from WhiB-family transcription factors, cAMP signaling, PhoPR, TrcPR and KdpDE two-component signaling, which could coordinately mediate transcriptional responses during starvation (**Supplementary Fig. 2b; Supplementary Table 3**). Overall, RNA-seq evidenced global transcriptional reprogramming, underscoring the value of investigating multiple levels of gene regulation when investigating the adaptation of bacterial pathogens to physiological or chemotherapeutic stress.

***Proteomics suggests a diminished contribution of leucine and lysine catabolism to ketogenesis in starved persisters.*** Of the two possible pathways for ketone genesis, KEGG pathway enrichments (**Supplementary Fig. 3c**) for significantly upregulated (>1.3-fold) proteins during starvation days 4 and 20 (S4, S20) and resuscitation day 6 (R6) did not identify significant changes in *leucine and lysine degradation* or metabolism but on the other hand picked out *fatty-acid metabolism*, as perturbed pathways

during starvation, this suggests that ketogenic amino acid metabolism has a limited contrition to total ketone body formation in mycobacteria.

***Examining the interplay between $H_2 0_2$ hyper-susceptibility and heme iron in starved persisters***. Given the observed connections between, β-hydroxybutyrate and cholesterol metabolism (Figure 2-3), ROS production (Figure 5) and electron transport chain complexes (Supplementary Figure 4b) and CYP expression (Supplemental Tables 2-3), our investigation aimed to elucidate the impact of iron, specifically heme availability on ketone body metabolism and the survival of starved persisters. Preliminary investigations utilizing three azole-based heme inhibitors—clotrimazole, econazole, and miconazole—revealed the involvement of heme in starved persisters, as evidenced by retained sensitivity to the inhibitors (Supplementary Fig. 10a). Notably, the starved persisters exhibited sensitivity comparable to log-phase cells, despite their resistance to rifampicin, streptomycin, ethambutol and isoniazid (Figure 1f). Our exploration aligns with broader research indicating the critical role of iron metabolism in mycobacteria. Specifically, limiting Mtb's access to iron using an iron chelator was found to result in the dysregulation of cholesterol catabolism[8,9]. Furthermore, the correlation between iron overload in macrophages and an increased risk of tuberculosis (TB) with potential exacerbation of TB outcomes underscores the intricate connections between iron availability, cholesterol metabolism, and the survival strategies of Mtb in host macrophages[10,11].

One possibility is that while $H_2O_2$ can serve as an electron donor to drive CYP activity by generating Cpd 0 directly from the ferric enzyme via the peroxidise shunt (Supplementary Figure 10b), the CYP heme group and catalytic cysteine residues are exquisitely susceptible to $H_2O_2$ inactivation by Fe-mediated reduction that generates damaging hydroxyl radicals or ferryl-oxo species (i.e., Fenton chemistry)[12]. This latter mechanism for CYP inactivation is plausible since starved persisters exhibit all the conditions for Haber-Weiss and Bray-Gorin catalysis (acidic pH , continued ferric and ferrous ions retention, as suggested by sustained IdeR and increased MmpL4 and MmpL5 levels, and superoxide

generation) except one: $H_2O_2$[13]. The addition of $H_2O_2$ in this instance would serve to decouple the tightly linked iron and carbon metabolism pathways required for survival. Together, these findings contribute to our understanding of the multifaceted factors influencing mycobacterial persistence and underscore the interconnectedness of metabolic pathways during this process.

## Supplementary Figures

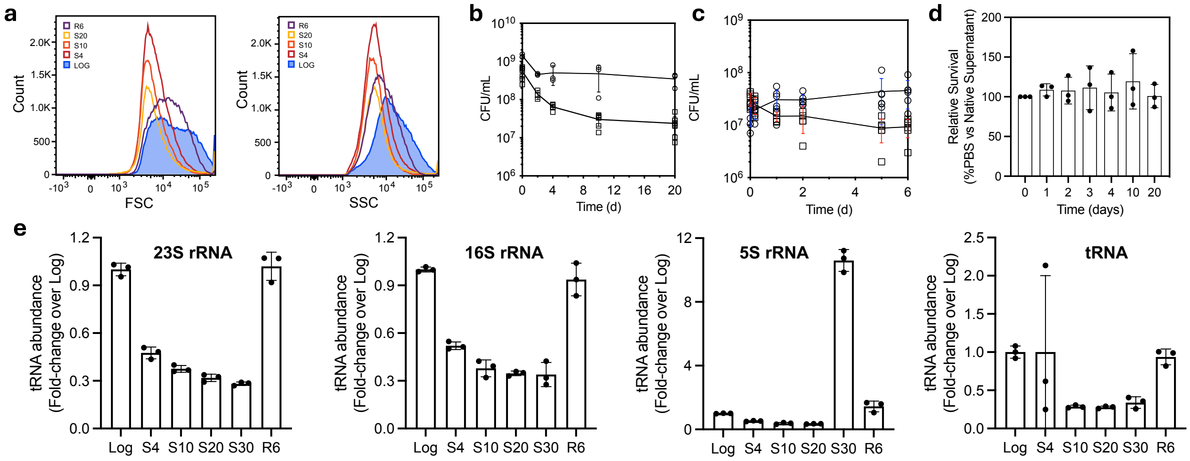


**Supplementary Figure 1.** Physiological and molecular features of mycobacterial starvation and persistence. (**a**) Comparison of FSC (left) and SSC (right) histogram profile underscorering starvation (S4, S10, S20), resuscitation (R6), and log growth shows starvation in PBS decreases the heterogeneity of both FSC and SSC profiles indicating smaller overall cell size and reduced cellular complexity. (**b, c**) Survival curves of BCG (**b**) and SMG (**c**) starved in $Mg^{2+}$ and $Ca^{2+}$ ion-containing DPBS (circles) or PBS (squares). For BCG, PBS data also presented in **Figure 1**; n = 6; $p < 0.01$; maximum cumulative D = 0.52 (two-sample Kolmogorov-Smirnov test). For SMG, n = 6; $p < 0.001$; maximum cumulative D = 0.77; two-sample Kolmogorov-Smirnov test. (**d**) CFU ratios of BCG recovered from 20-day cultures and re-starved for 20 days in fresh PBS versus native supernatant. N = 6; $p > 0.05$; one-way ANOVA with Tukey's HSD. (**e**) Intracellular levels of 23S, 16S, and 5S rRNA and tRNA in BCG at Log, S4, S10, S20, and R6 (n = 3; * $p < 0.05$; one-way ANOVA with Dunnett's test vs Log). See **Supplementary Data 4** for source data used in these graphs.

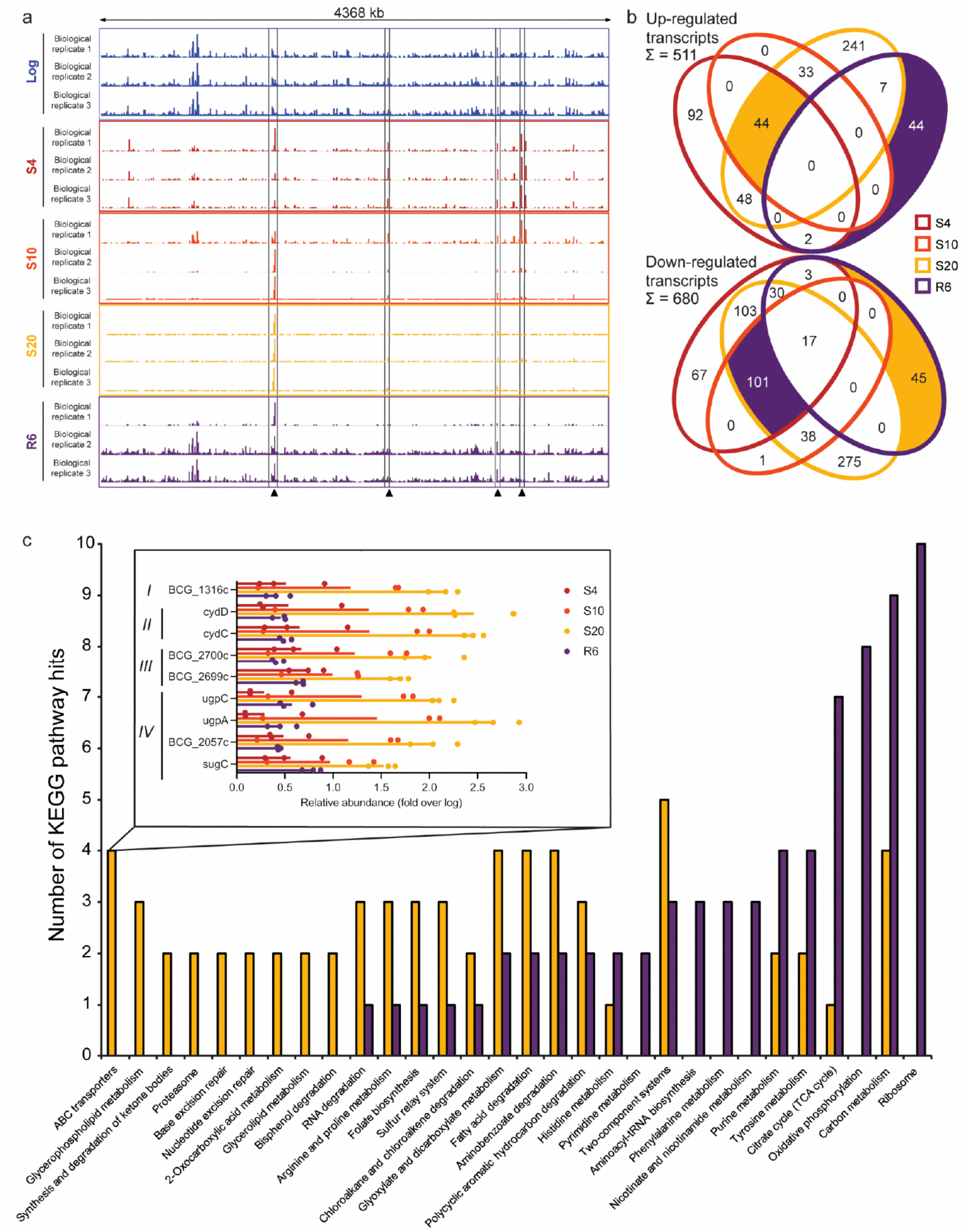
a
4368 kb
Log
S4
S10
S20
R6
Biological replicate 1
Biological replicate 2
Biological replicate 3
b
Up-regulated transcripts
Σ = 511
Down-regulated transcripts
Σ = 680
S4
S10
S20
R6
c
Number of KEGG pathway hits
I
II
III
IV
BCG_1316c
cydD
cydC
BCG_2700c
BCG_2699c
ugpC
ugpA
BCG_2057c
sugC
Relative abundance (fold over log)
ABC transporters
Glycerophospholipid metabolism
Synthesis and degradation of ketone bodies
Proteasome
Base excision repair
Nucleotide excision repair
2-Oxocarboxylic acid metabolism
Glycerolipid metabolism
Bisphenol degradation
RNA degradation
Arginine and proline metabolism
Folate biosynthesis
Sulfur relay system
Chloroalkane and chloroalkene degradation
Glyoxylate and dicarboxylate metabolism
Fatty acid degradation
Aminobenzoate degradation
Polycyclic aromatic hydrocarbon degradation
Histidine metabolism
Pyrimidine metabolism
Two-component systems
Aminoacyl-tRNA biosynthesis
Phenylalanine metabolism
Nicotinate and nicotinamide metabolism
Purine metabolism
Tyrosine metabolism
Citrate cycle (TCA cycle)
Oxidative phosphorylation
Carbon metabolism
Ribosome

**Supplementary Figure 2.** RNA-seq and differential pathway analysis of the mycobacterial transcriptome during starvation. (**a**) Visualization of RNA-seq coverage across the BCG genome. Each sequencing track represents the one biological replicate consisting of 2 appended technical replicate runs. Peaks heights indicate read coverage. Arrows (bottom from left to right) highlight the prominent transcripts from 16S-23S-5S rRNA, *rnpB*, *ssr* and *rsbW-usfY,* respectively. Note the gradual reduction in RNA signal intensity during the starvation time course and restoration of RNA levels during resuscitation in nutrient-replete medium. (**b**) Venn diagram of significantly up- and down-regulated genes in S4, S10, S20 and R6 vs Log cultures (>1.5-fold change; $p < 0.05$; De-seq multi-testing). A total of 680 down-regulated transcripts and 511 up-regulated transcripts fulfilled the criteria. The individual and overlapping areas represent the number of uniquely or co-expressed transcripts at the indicated time points. Genes down-regulated at R6 but up-regulated during starvation (S4, S10 and S20) are core set 1 (yellow shading). Conversely, genes up-regulated at R6 but down-regulated during starvation are core set 2 (purple shading). (**c**) Pathway analysis of core gene sets 1 and 2 showing canonical pathways (KEGG annotation) modulated by the genes with differential expression between R6 and starved cultures (S4, S10 and S20). Specificity was determined by selecting pathways that fit the condition:

$$\frac{core\,set\,1\,hits}{core\,set\,2\,hits} \geq 1.5\,or\,\frac{core\,set\,2\,hits}{core\,set\,1\,hits} \geq 1.5$$

Purple bars (core set 1) and yellow bars (core set 2). *Insert*: Example of pathway enrichment with ABC transporters. Four groups of hits were identified. *(I)* multidrug-efflux transporter *BCG_1316c; (II)* ATP-binding cassette subfamily C (ABCC), cytochrome biosynthesis permeases cydC and cydD; (*III)* Fluoroquinolones transport system permease proteins *BCG_2699c* and *BCG_2700c* (MTB *Rv2686c* and *Rv2687c* homologs); and (*IV)* Multiple sugar transporters (*sugC* and *BCG_2057c*) and *sn*-glycerol 3-phosphate transporters (*ugpA* and *ugpC*). Transcripts from all aforementioned genes possess similar patterns of expression (**Supplementary Data 1**). See **Supplementary Data 4** for source data used in these graphs.

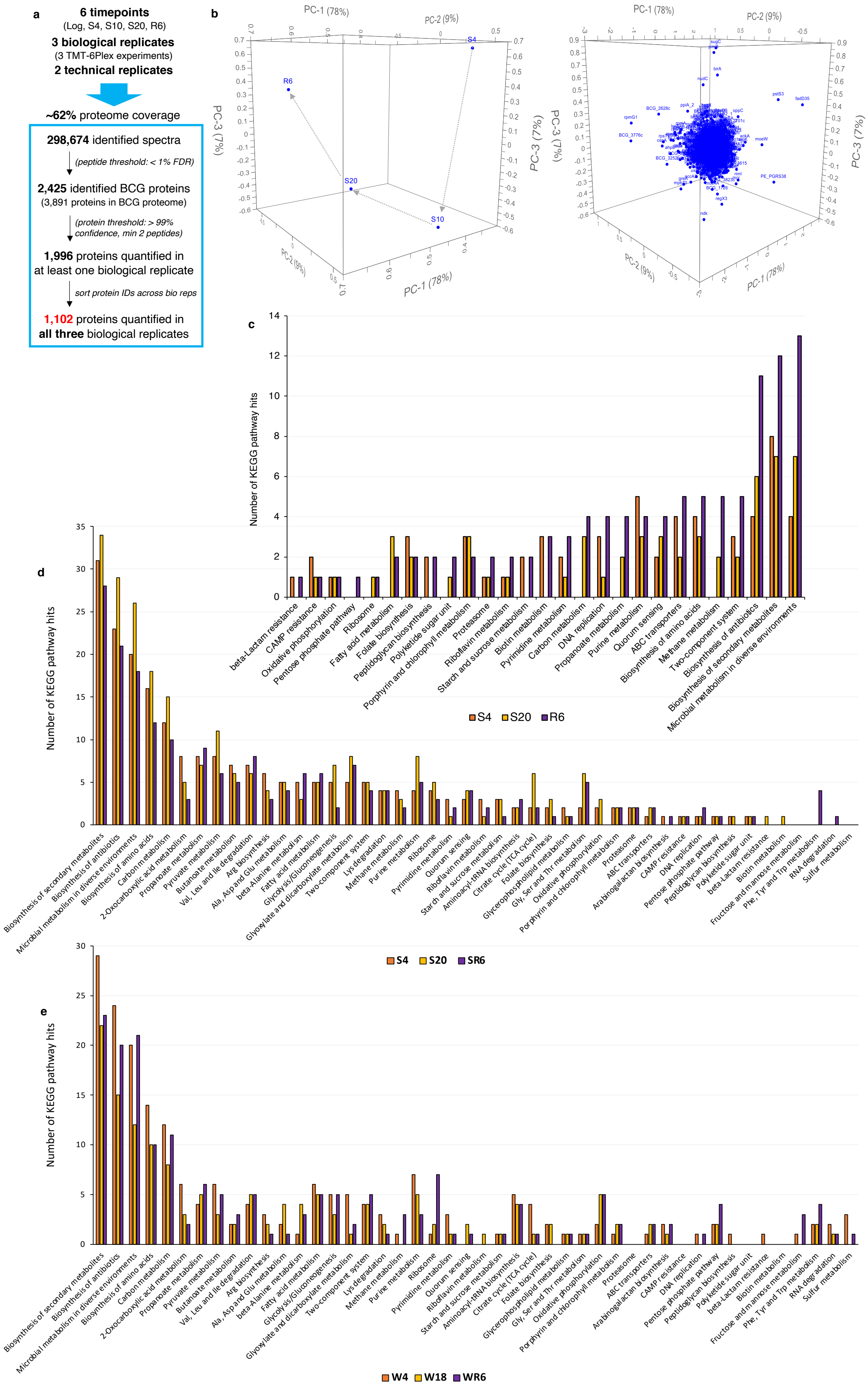
a
6 timepoints
(Log, S4, S10, S20, R6)
3 biological replicates
(3 TMT-6Plex experiments)
2 technical replicates
~62% proteome coverage
298,674 identified spectra
(peptide threshold: < 1% FDR)
2,425 identified BCG proteins
(3,891 proteins in BCG proteome)
(protein threshold: > 99% confidence, min 2 peptides)
1,996 proteins quantified in at least one biological replicate
sort protein IDs across bio reps
1,102 proteins quantified in all three biological replicates
b
PC-1 (78%)
PC-2 (9%)
PC-3 (7%)
S4
R6
S20
S10
c
Number of KEGG pathway hits
beta-Lactam resistance
CAMP resistance
Oxidative phosphorylation
Pentose phosphate pathway
Ribosome
Fatty acid metabolism
Folate biosynthesis
Peptidoglycan biosynthesis
Polyketide sugar unit
Porphyrin and chlorophyll metabolism
Proteasome
Riboflavin metabolism
Starch and sucrose metabolism
Biotin metabolism
Pyrimidine metabolism
Carbon metabolism
DNA replication
Propanoate metabolism
Purine metabolism
Quorum sensing
ABC transporters
Biosynthesis of amino acids
Methane metabolism
Two-component system
Biosynthesis of antibiotics
Biosynthesis of secondary metabolites
Microbial metabolism in diverse environments
S4 S20 R6
d
Number of KEGG pathway hits
S4 S20 SR6
e
Number of KEGG pathway hits
W4 W18 WR6

**Supplementary Figure 3.** Quantitative proteomics depicts dynamic functional gene regulation during starvation-induced NRP. (**a**) Summary statistics of quantitative proteomic coverage of BCG genome by tandem mass tag (TMT) 6-Plex mass spectrometry (UniProt, *M. bovis* BCG Pasteur 1173P2 MYCBP). (**b**) Principal component analysis of proteomics time course data. The scores plot (left) shows subsets of proteins are strongly distinguishing each time point in the starvation time course. (**c**) KEGG pathway enrichments for significantly upregulated (>1.3-fold) proteins during starvation days 4 and 20 (S4, S20) and resuscitation day 6 (R6) underscore phenotypic differences between early (S4) and late (S20) non-replicating persistence and resuscitation (R6). See **Supplementary Data 2** for the complete set of starvation proteomics data. (**d, e**) KEGG pathway enrichments for significantly upregulated (>1.3-fold) proteins in a mean-normalized dataset of 379 mutually quantified proteins in *M. bovis* BCG subjected to starvation (**d**) and hypoxia (**e**). See **Supplementary Data 4** for source data used in these graphs.

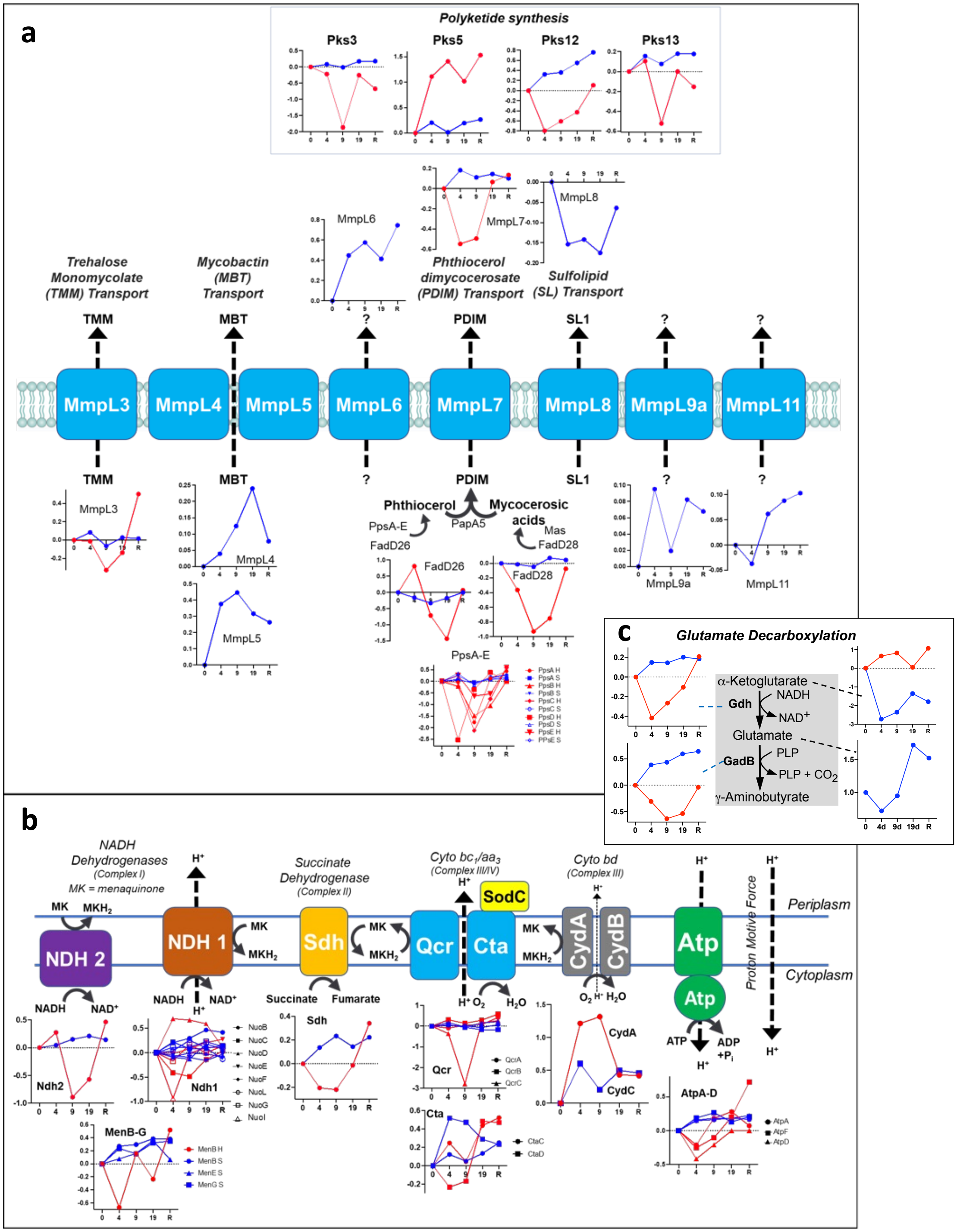


**Supplementary Figure 4.** (**a**) Cell wall synthesis factors, (**b**) electron transport pathways, and glutamate decarboxylation during BCG starvation and hypoxia. Graphs were prepared from data in **Supplementary Data 2**, with protein $\log_2$(fold-change) vs days of hypoxia (red) or starvation (blue) plotted. Each datapoint represents the average of triplicate cultures. See **Supplementary Data 4** for source data used in these graphs.

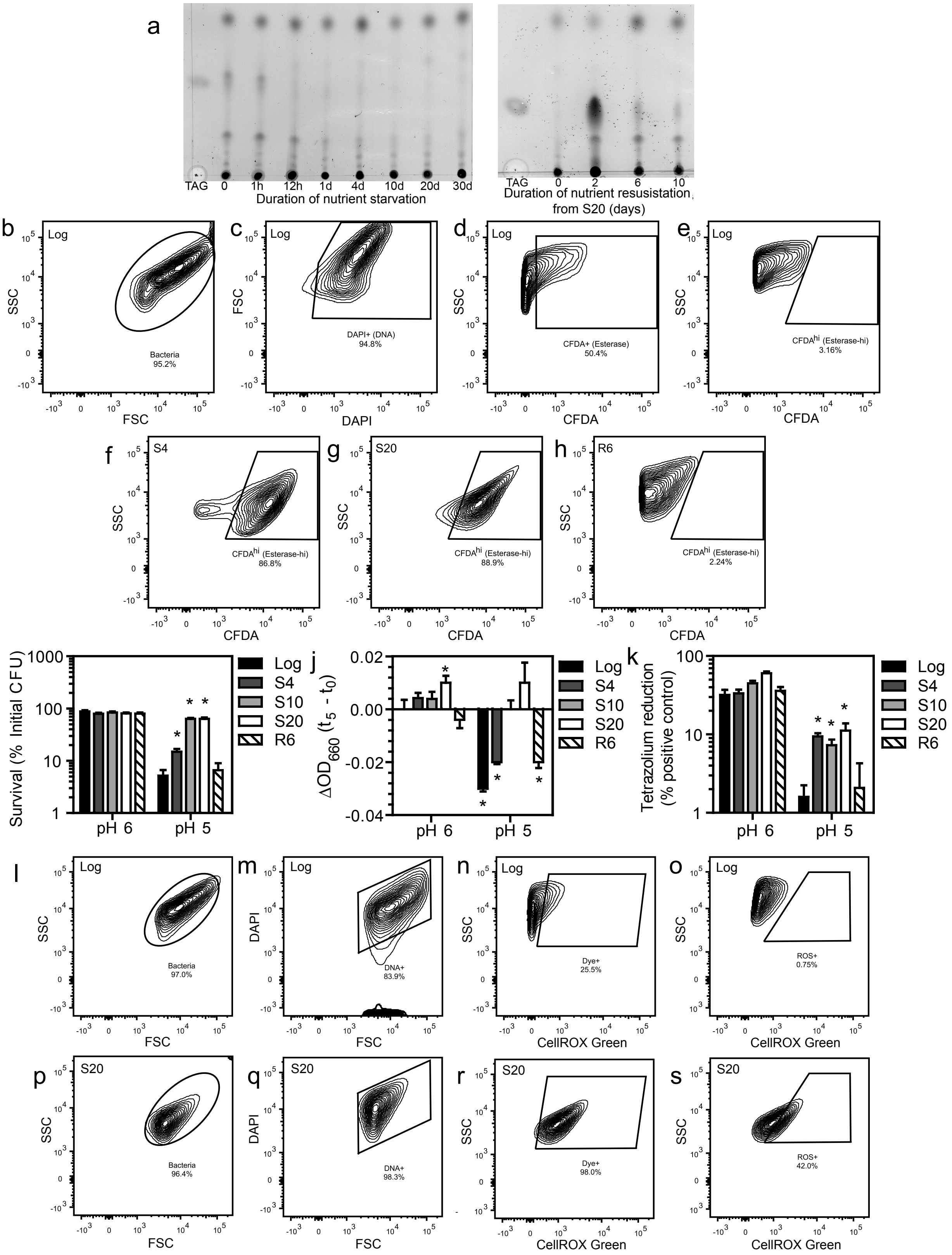

a
TAG 0 1h 12h 1d 4d 10d 20d 30d
Duration of nutrient starvation
TAG 0 2 6 10
Duration of nutrient resusistation, from S20 (days)
b Log SSC FSC Bacteria 95.2%
c Log FSC DAPI DAPI+ (DNA) 94.8%
d Log SSC CFDA CFDA+ (Esterase) 50.4%
e Log SSC CFDA CFDAhi (Esterase-hi) 3.16%
f S4 SSC CFDA CFDAhi (Esterase-hi) 86.8%
g S20 SSC CFDA CFDAhi (Esterase-hi) 88.9%
h R6 SSC CFDA CFDAhi (Esterase-hi) 2.24%
i Survival (% Initial CFU) pH 6 pH 5 Log S4 S10 S20 R6
j ΔOD660 (t5 - t0) pH 6 pH 5 Log S4 S10 S20 R6
k Tetrazolium reduction (% positive control) pH 6 pH 5 Log S4 S10 S20 R6
l Log SSC FSC Bacteria 97.0%
m Log DAPI FSC DNA+ 83.9%
n Log SSC CellROX Green Dye+ 25.5%
o Log SSC CellROX Green ROS+ 0.75%
p S20 SSC FSC Bacteria 96.4%
q S20 DAPI FSC DNA+ 98.3%
r S20 SSC CellROX Green Dye+ 98.0%
s S20 SSC CellROX Green ROS+ 42.0%

**Supplementary Figure 5.** Lipid utilization and consequences during starvation. (**a**) Starvation-induced changes in lipid levels in BCG. Representative thin-layer chromatograms (TLCs) of starved (left panel) and resuscitated (right panel) BCG at indicated time points (TAG: Triacylglycerol standard; d: days). (**b-h**) Starvation-induced changes in lipid esterase activity. Contour plots depicting gating scheme used to identify cells with elevated esterase activity ($CFDA^{hi}$) by flow cytometry. Cells within the "bacteria" gate (**b**) were gated for DNA presence indicated by DAPI staining (**c**). Cells were further gated for uptake of CFDA (CDFA+, **d**) or esterase activity ($CFDA^{hi}$, **e**) followed by a threshold set for basal activity in Log. (**f-h**) Representative CFDA contour plots for S4 (**f**), S20 (**g**), and R6 (**h**) depict shifts in esterase activity during starvation. (**i-k**) Starvation-induced tolerance to acidic pH reflecting adaptation to ketoacidotic intracellular conditions. Growth phenotypes and metabolic activities of BCG during log growth, days of starvation (S4, S10, S20), and resuscitation (R6) in media buffered at pH 5 and 6. Viability was measured by CFU (**i**), change in $OD_{600}$ (**j**), and reductive respiratory potential (tetrazolium reduction, **k**) after 5 d of exposure to media at pH 5 or 6. Data represent mean ±SD for N = 3; * $p < 0.05$; one-way ANOVA with Dunnett's Multiple Comparison. (**l-s**) Starvation-induced changes in ROS levels. Contour plots of Log and S20 BCG depicting gating scheme used to identify ROS-producing cells ($CellROX^{hi}$). Cells in the "bacteria" gate (**l**) were gated for DNA content indicated by DAPI staining (**m**). Cells with DNA were further identified as having intracellular CellROX dye (**n**) and ROS-activated CellROX ($CellROX^{hi}$, **o**). $CellROX^{hi}$ cells were designated as those with fluorescence greater than basal levels. See **Supplementary Data 4** for source data used in these graphs.

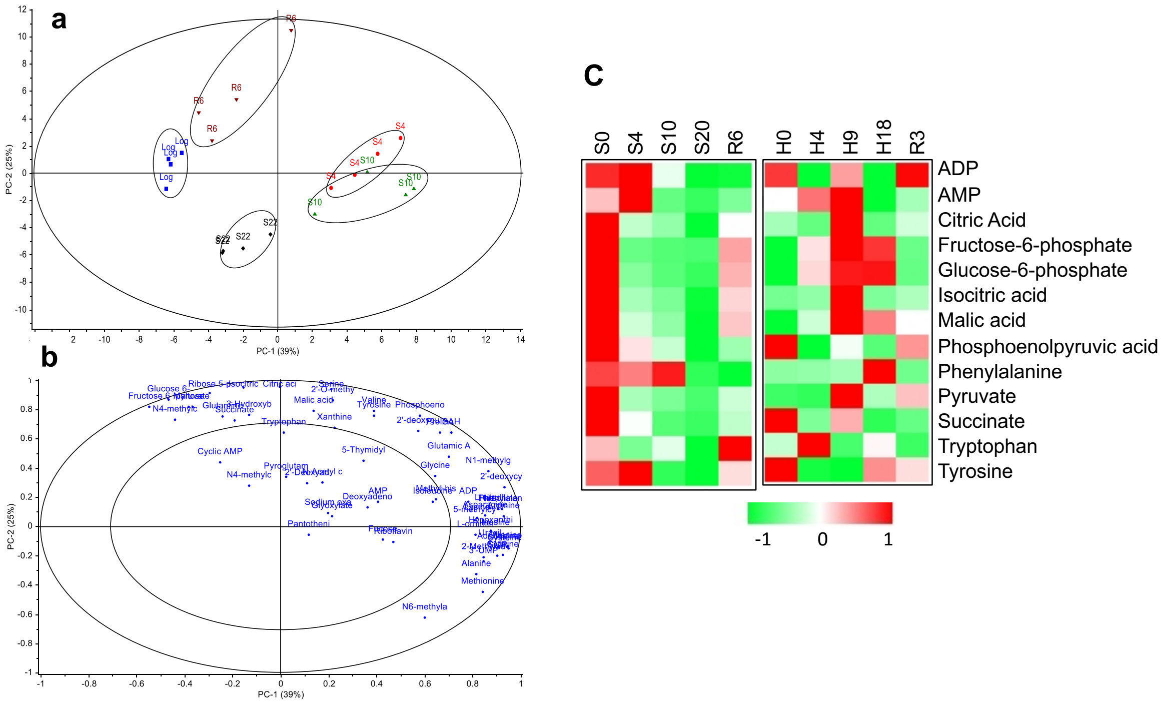


**Supplementary Figure 6:** Multivariate statistical analysis of quantitative metabolomics emphasizes metabolic plasticity during starvation and distinct shifts in metabolism in non-replicative, persistent states of BCG subjected to hypoxia and starvation. Scores (**a**) and loadings (**b**) plots from a principal component analysis of metabolites quantified in *M. bovis* BCG in log growth in rich medium (Log, blue) and in BCG subjected to starvation for 4 (S4, brown), 10 (S10, orange), and 20 (S20, yellow) days and then resuscitation in nutrient-replete medium for 6 days (R6, purple). The plot shows that subsets of metabolites strongly distinguish early (S4), late (S10, S20), and nutrient-replete states (Log and R6). (**c**) Hierarchical clustering of data for metabolites measured in common for hypoxia (right; H0: aerobic; H4, H9, H18: 4, 9 and 18 days of hypoxia; R3: 3 days of aerobic growth) and starvation (as in panels **a,b**). The results show up-regulated glycolysis metabolites in hypoxia and down-regulation in starvation, which is consistent with the shift to β-oxidation of fatty acids in hypoxia. Data are taken from **Supplementary Data 3**. See **Supplementary Data 4** for source data used in these graphs.

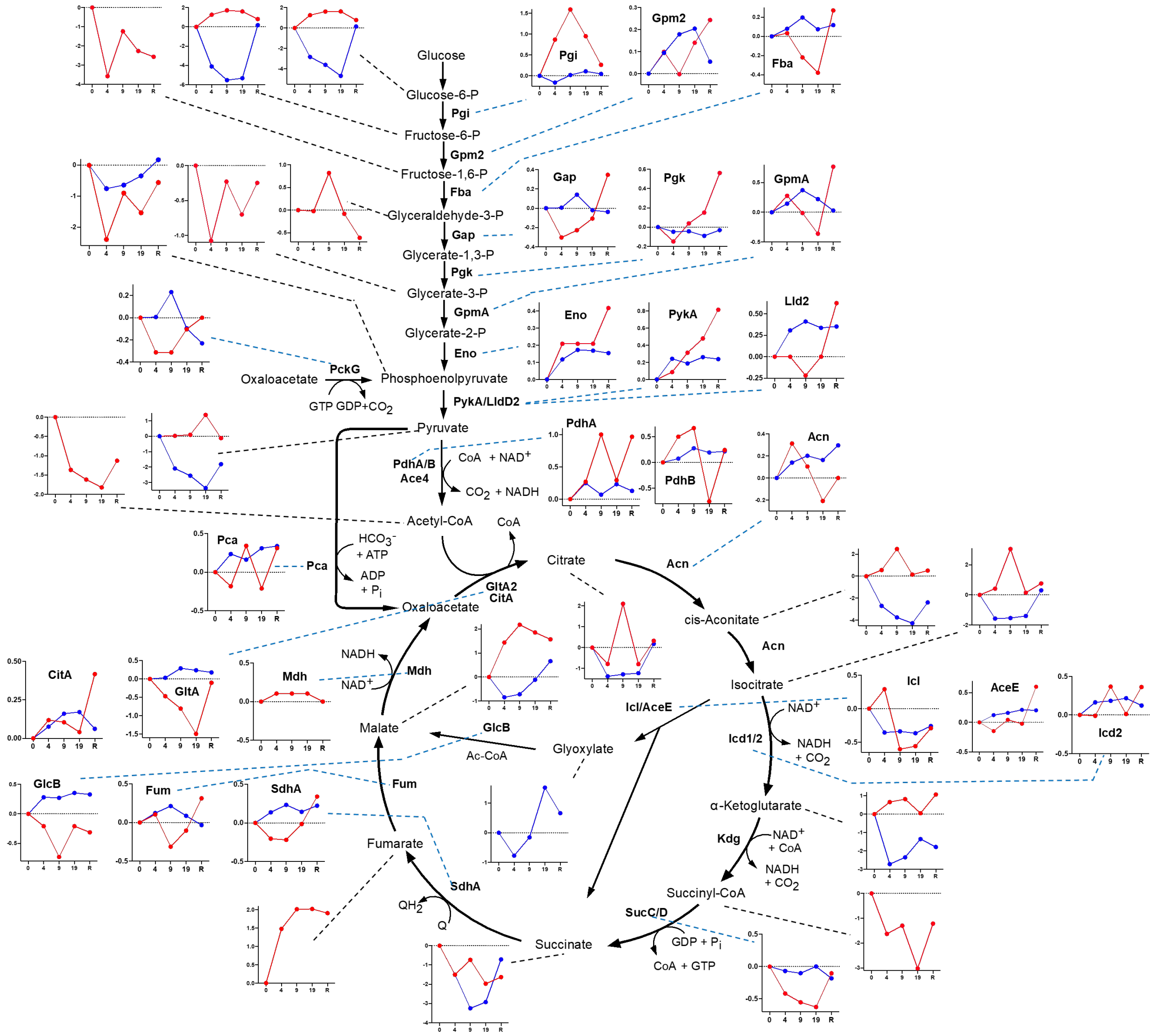


**Supplementary Figure 7.** Changes in the glycolysis and citrate cycle pathways during starvation and hypoxia. Protein (names on graphs) or metabolite (unlabeled graphs) $log_2$(fold-change) vs days of hypoxia (red) or starvation (blue) plotted with each datapoint representing the average of triplicate cultures. Data are derived from **Supplementary Data 2** and **3**. See **Supplementary Data 4** for source data used in these graphs.

***Fold Change [Protein] vs [mRNA]***

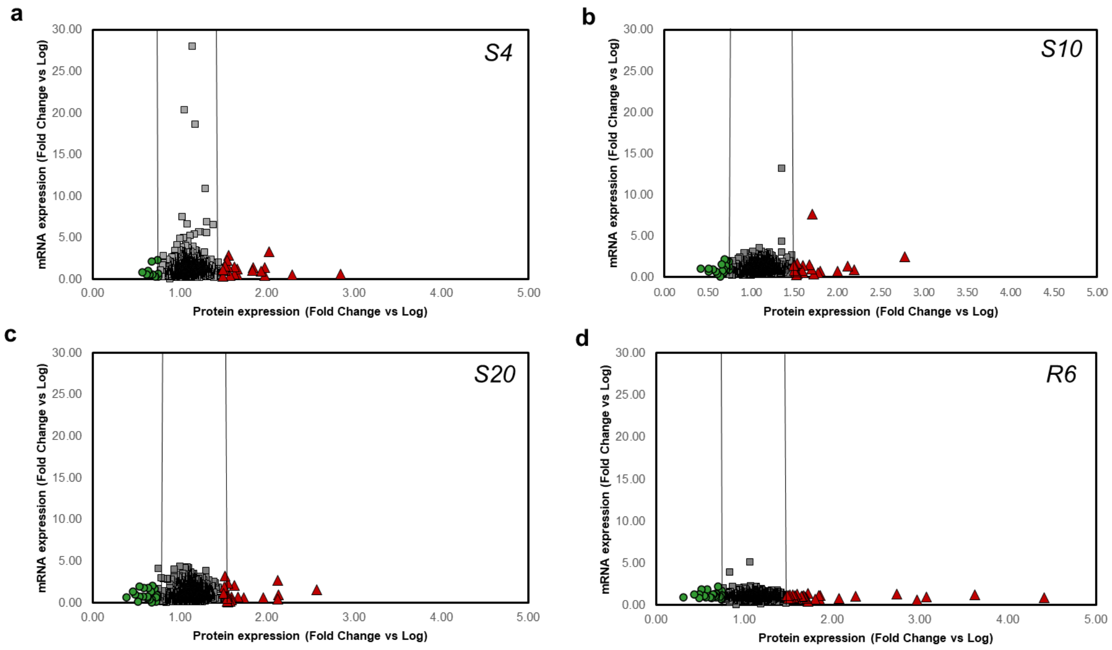


**Supplementary Figure 8**. Scatterplots for protein against mRNA expression for 1170 genes classified by timepoint at (**a**) S4, (**b**) S10, (**c**) S20 and (**d**) R6. Mean non-normalized mRNA and protein fold changes against abundances at Log are shown (N=3). For visualization, red triangles: >1.5 fold increase relative to Log; green circles: < 0.75 fold decrease relative to Log. Data from **Supplementary Data 1** and **2**. See **Supplementary Data 4** for source data used in these graphs.

**Supplementary Figure 9.**

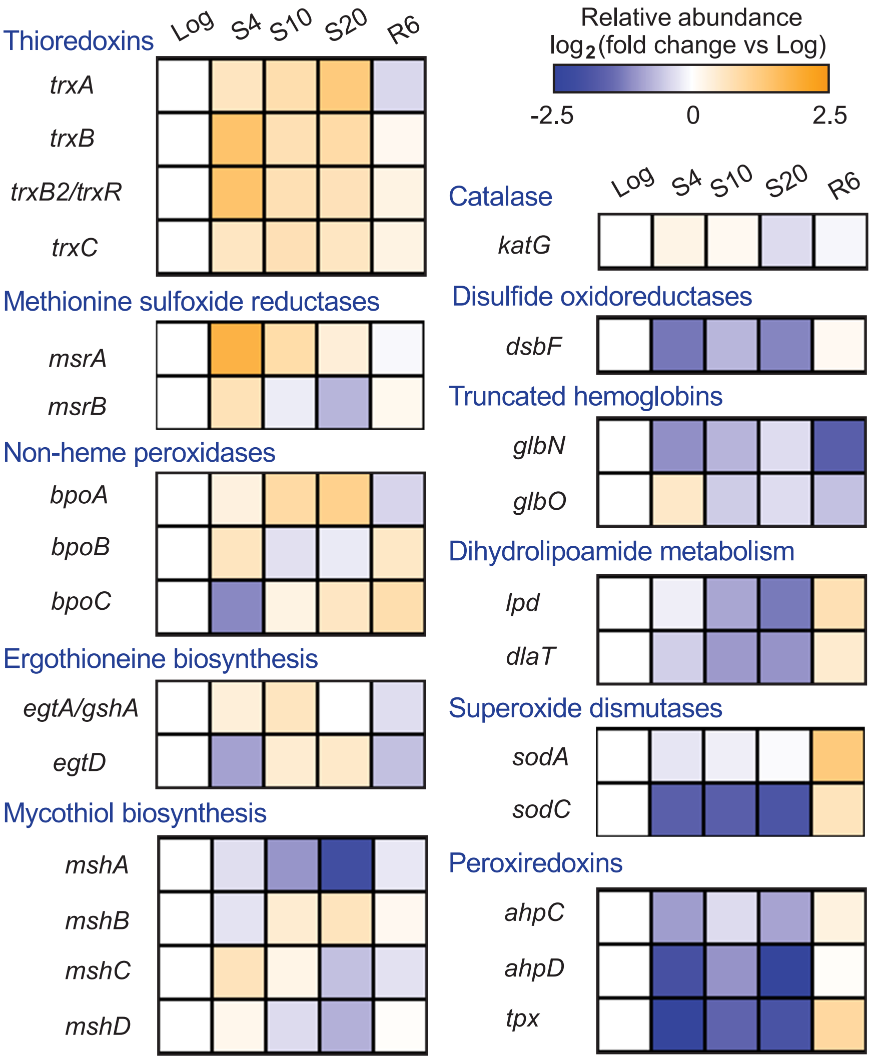


**Supplementary Figure 9.** Expression heat maps of antioxidant defense genes from RNA-seq. For visualization, transcript expression at each time point is represented by a colored square along a blue-orange scale (N=3). Unlike the heatmaps in **Figures 4** and **5b**, unnormalized values (not z-score) are shown to depict abundances relative to Log (white squares) with no reduction in dimensionality. Data were taken from **Supplementary Data 1**. See **Supplementary Data 4** for source data used in these graphs.

**a**

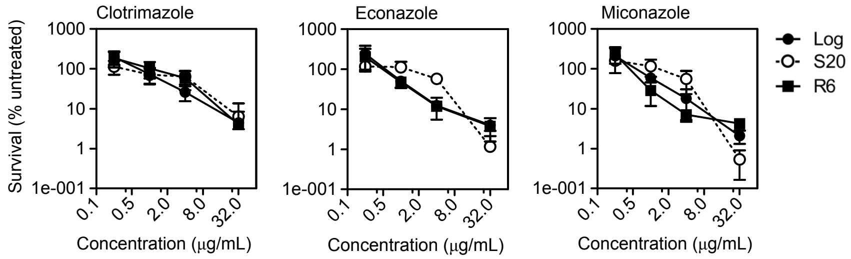


**b**

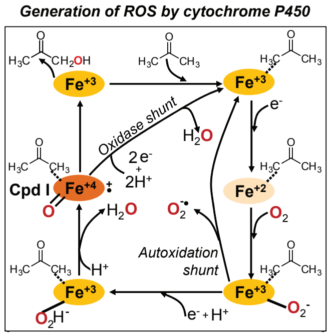


**Supplementary Figure 10.** (a) Survival of Log, S20 and R6 BCG to azole-based CYP inhibitors clotrimazole, econazole, and miconazole. (b) Mechanism for heme-mediated generation of ROS by CYPs. Cultures were treated for 48 h at the indicated concentrations. Data represent mean ± SD for n ≥ 3. See **Supplementary Data 4** for source data used in these graphs.

## Supplementary Tables

**Supplementary Table 1.** Changes in forward and side scatter light during starvation.

| Parameter | Log [a] | S20 [a] | S20R6 [a] |
|---|---|---|---|
| Median FSC | 15862 (1252) | 5420 (129) | 14023 (249) |
| Robust CV of FSC (interquartile range) | 182 (14) | 75 (6) | 140 (13) |
| Median SSC | 13899 (857) | 5283 (264) | 8967 (486) |
| Robust CV of SSC (interquartile range) | 117 (8) | 61 (3) | 102 (4) |

[a] mean (SD), N = 3 biological replicates from mean of 4 technical replicates

**Supplementary Table 2.** Changes in metabolism (tetrazolium reduction 485 nm) when BCG from log, S4, S10, S20, and S20R6 were transferred to single carbon source or chemical.

**Supplementary Table 4**: Changes in metabolism (tetrazolium reduction 485 nm) when BCG from log, S4, S10, S20, and S20R6 were transferred to single carbon source or chemical. Data represent mean ± SEM.

| Carbon source | Log | S4 | S10 | S20 | R6 |
|---|---|---|---|---|---|
| Full media | 0.057 ± 0.006 | 0.071 ± 0.002 | 0.079 ± 0.006 | 0.101 ± 0.006 | 0.061 ± 0.004 |
| Dextrin | 0.006 ± 0.001 | 0.014 ± 0.006 | 0.06 ± 0.006 | 0.061 ± 0.011 | 0.006 ± 0.003 |
| D-Maltose | 0.001 ± 0.001 | 0 | 0 | 0 | 0 |
| D-Trehalose | 0.085 ± 0.043 | 0.099 ± 0.004 | 0.051 ± 0.016 | 0.020 ± 0.004 | 0.110 ± 0.011 |
| D-Cellobiose | 0 | 0 | 0 | 0.002 ±0 | 0.002 ± 0.002 |
| Gentiobiose | 0.005 ± 0.002 | 0 | 0.014 ± 0.006 | 0.021 ± 0.003 | 0.008 ± 0.002 |
| Sucrose | 0 | 0 | 0.001 ±0 | 0.004 ±0 | 0 |
| D-Turanose | 0 | 0 | 0 | 0 | 0 |
| Stachyose | 0.122 ± 0.028 | 0 | 0 | 0 | 0 |
| D-Raffinose | 0 | 0 | 0 | 0.002 ± 0.001 | 0 |
| α-D-Lactose | 0.004 ± 0.004 | 0 | 0.004 ± 0.004 | 0.025 ± 0.023 | 0 |
| D-Melibiose | 0.002 ± 0.002 | 0 | 0.014 ± 0.007 | 0.023 ± 0.004 | 0.002 ± 0.001 |
| β-Methyl-D-glucoside | 0 | 0 | 0 | 0.001 ± 0.001 | 0.000 ±0 |
| D-Salicin | 0.026 ± 0.016 | 0 | 0 | 0 | 0.036 ± 0.004 |
| N-Acetyl-D-glucosamine | 0.001 ± 0.001 | 0 | 0 | 0 | 0 |
| N-Acetyl-D-mannosamine | 0 | 0 | 0 | 0.003 ± 0.001 | 0 |
| N-Acetyl-D-galactosamine | 0 | 0 | 0.003 ±0 | 0.003 ±0 | 0 |
| N-Acetyl-neuraminic acid | 0 | 0 | 0 | 0 | 0 |
| α-D-Glucose | 0 | 0 | 0.005 ± 0.001 | 0.007 ± 0.001 | 0 |
| D-Mannose | 0 | 0 | 0.008 ± 0.005 | 0.008 ± 0.002 | 0 |
| D-Fructose | 0.023 ± 0.006 | 0.028 ± 0.003 | 0.045 ± 0.010 | 0.052 ± 0.008 | 0.021 ±0 |
| D-Galactose | 0.008 ± 0.003 | 0.010 ± 0.003 | 0.032 ± 0.013 | 0.023 ± 0.001 | 0.020 ± 0.003 |
| 3-Methyl-glucose | 0.006 ± 0.004 | 0 | 0.014 ± 0.004 | 0.007 ± 0.001 | 0 |
| D-Fucose | 0.012 ± 0.002 | 0.009 ± 0.001 | 0.032 ± 0.003 | 0.029 ± 0.001 | 0.018 ± 0.003 |
| L-Fucose | 0.050 ± 0.003 | 0.036 ± 0.001 | 0.066 ± 0.002 | 0.066 ± 0.005 | 0.055 ± 0.002 |
| L-Rhamnose | 0 | 0 | 0.030 ± 0.006 | 0.024 ±0 | 0 |
| Inosine | 0 | 0 | 0 | 0 | 0 |
| D-Sorbitol | 0 | 0 | 0.003 ± 0.002 | 0.004 ±0 | 0 |
| D-Mannitol | 0 | 0 | 0.010 ± 0.007 | 0.005 ± 0.002 | 0 |
| D-Aabitol | 0 | 0.009 ± 0.002 | 0.009 ± 0.006 | 0.004 ± 0.001 | 0 |
| Myo-inositol | 0 | 0 | 0.013 ± 0.005 | 0.004 ± 0.003 | 0 |
| Glycerol | 0.243 ± 0.012 | 0.304 ± 0.013 | 0.119 ± 0.011 | 0.018 ± 0.006 | 0.324 ± 0.011 |
| D-Glucose-6-phosphate | 0 | 0 | 0.015 ± 0.002 | 0.014 ± 0.002 | 0 |
| D-Fructose-6-phosphate | 0.081 ± 0.006 | 0.087 ± 0.001 | 0.103 ±0 | 0.102 ± 0.005 | 0.068 ± 0.004 |
| D-Aspartic acid | 0 | 0 | 0.017 ± 0.006 | 0 | 0 |
| D-Serine | 0 | 0 | 0 | 0 | 0 |
| Gelatin | 0 | 0 | 0 | 0 | 0 |
| Glycyl-L-proline | 0 | 0.002 ± 0.002 | 0.017 ± 0.009 | 0.001 ± 0.001 | 0.001 ± 0.001 |
| L-Alanine | 0 | 0 | 0 | 0 | 0 |
| L-Arginine | 0 | 0 | 0 | 0 | 0 |
| L-Aspartic acid | 0.004 ± 0.003 | 0.031 ± 0.007 | 0.020 ± 0.005 | 0 | 0 |
| L-Glutamic acid | 0.023 ± 0.007 | 0.082 ± 0.008 | 0.050 ± 0.012 | 0 | 0.020 ± 0.004 |
| L-Histidine | 0 | 0 | 0.005 ± 0.001 | 0.010 ±0 | 0 |
| L-Pyroglutamic acid | 0 | 0.007 ± 0.003 | 0.019 ± 0.003 | 0 | 0 |
| L-Serine | 0 | 0 | 0.001 ± 0.001 | 0 | 0 |
| Pectin | 0 | 0.001 ±0 | 0.015 ± 0.002 | 0.016 ± 0.002 | 0 |
| D-Galacturonic acid | 0.030 ± 0.009 | 0.054 ± 0.002 | 0.078 ± 0.010 | 0.080 ± 0.013 | 0.025 ± 0.002 |
| L-Galactonic acid lactone | 0 | 0 | 0 | 0 | 0 |
| D-Gluconic acid | 0 | 0 | 0.024 ± 0.003 | 0 | 0.011 ± 0.008 |
| D-Glucuronic acid | 0.05 ± 0.004 | 0.113 ± 0.004 | 0.135 ± 0.010 | 0.134 ± 0.019 | 0.056 ± 0.005 |
| Glucuronamide | 0.148 ± 0.004 | 0.153 ± 0.005 | 0.177 ± 0.018 | 0.175 ± 0.002 | 0.140 ± 0.001 |
| Muric acid | 0 | 0 | 0.003 ± 0.001 | 0 | 0 |
| Quinic acid | 0 | 0 | 0.018 ± 0.004 | 0 | 0 |
| D-Saccharic acid | 0 | 0 | 0 | 0 | 0 |
| p-Hydroxy-phenylacetate | 0 | 0 | 0 | 0 | 0 |
| Methyl pyruvate | 0 | 0.058 ± 0.017 | 0.037 ± 0.022 | 0.003 ± 0.002 | 0 |
| D-lactic acid methyl ester | 0 | 0.021 ± 0.001 | 0.029 ± 0.007 | 0 | 0.011 ± 0.008 |
| L-Lactic acid | 0.003 ± 0.003 | 0.030 ± 0.003 | 0.018 ± 0.009 | 0 | 0.019 ± 0.006 |
| Citric acid | 0.023 ± 0.008 | 0.046 ± 0.006 | 0.049 ± 0.014 | 0.007 ± 0.002 | 0.026 ± 0.003 |
| α-Keto-glutaric acid | 0.060 ± 0.003 | 0.117 ± 0.004 | 0.162 ± 0.007 | 0.1 ± 0.005 | 0.074 ± 0.009 |
| D-Malic acid | 0 | 0 | 0.014 ± 0.001 | 0 | 0 |
| L-Malic acid | 0 | 0.018 ± 0.002 | 0.042 ± 0.001 | 0.001 ± 0.001 | 0.001 ± 0.001 |
| Bromo-succinic acid | 0 | 0.024 ± 0.001 | 0.029 ± 0.002 | 0 | 0.004 ± 0.003 |
| Tween 40 | 0.032 ± 0.006 | 0.29 ± 0.006 | 0.023 ± 0.005 | 0.013 ± 0.003 | 0.24 ± 0.003 |
| γ-Amino-butyric acid | 0 | 0.003 ±0 | 0.027 ± 0.008 | 0.002 ± 0.002 | 0.000 ±0 |
| α-Hydro-butyric acid | 0 | 0 | 0 | 0 | 0 |
| β-Hydro-D,L-butyric acid | 0 | 0.037 ± 0.002 | 0.040 ± 0.009 | 0 | 0.003 ± 0.001 |
| α-Keto-butyric acid | 0.019 ± 0.006 | 0.028 ± 0.011 | 0.046 ± 0.014 | 0.059 ± 0.005 | 0.018 ± 0.003 |
| Acetoacetic acid | 0.112 ± 0.003 | 0.149 ± 0.005 | 0.179 ± 0.016 | 0.147 ± 0.014 | 0.107 ± 0.013 |
| Propionic acid | 0.021 ± 0.003 | 0.082 ± 0.005 | 0.041 ± 0.004 | 0.017 ± 0.004 | 0.034 ± 0.004 |
| Acetic acid | 0.086 ± 0.031 | 0.251 ± 0.006 | 0.144 ± 0.004 | 0.042 ± 0.009 | 0.128 ± 0.012 |
| Formic acid | 0 | 0 | 0 | 0 | 0 |

**Supplementary Table 3.** Changes in growth (OD at 660 nm) when BCG from log, S4, S10, S20, and S20R6 were transferred to single carbon source or chemical.

| Carbon source | Log | S4 | S10 | S20 | R6 |
|---|---|---|---|---|---|
| Full media | 0.031 ± 0.007 | 0.036 ± 0.002 | 0.060 ± 0.006 | 0.081 ± 0.008 | 0.036 ± 0.006 |
| Dextrin | 0.005 ± 0.001 | (0.028) ± 0.002 | 0.027 ± 0.008 | 0.049 ± 0.012 | (0.028) ± 0.003 |
| D-maltose | (0.021) ± 0.003 | (0.040) ± 0.003 | (0.011) ± 0.007 | 0.001 ± 0.001 | (0.009) ± 0.003 |
| D-trehalose | 0.115 ± 0.060 | 0.157± 0.006 | 0.068 ± 0.021 | 0.027 ± 0.004 | 0.143 ± 0.016 |
| D-cellobiose | (0.020) ± 0.003 | (0.037) ± 0.003 | (0.010) ± 0.005 | 0.005 ±0 | (0.011) ± 0.007 |
| Gentiobiose | 0.010 ± 0.004 | (0.002) ± 0.001 | 0.022 ± 0.007 | 0.038 ± 0.002 | 0.012 ± 0.002 |
| Sucrose | (0.019) ± 0.005 | (0.029) ± 0.003 | 0.002 ±0 | 0.007 ± 0.001 | (0.006) ±0 |
| ±D-turanose | (0.055) ± 0.007 | (0.056) ± 0.002 | (0.023) ± 0.010 | (0.003) ± 0.001 | (0.045) ± 0.004 |
| Stachyose | 0.158 ± 0.048 | (0.031) ± 0.004 | (0.008) ± 0.003 | 0.000 ± 0.003 | (0.009) ± 0.002 |
| D-raffinose | (0.015) ± 0.004 | (0.023) ± 0.005 | (0.006) ± 0.003 | 0.004 ± 0.002 | (0.015) ± 0.001 |
| α-D-lactose | (0.014) ± 0.005 | (0.033) ± 0.003 | (0.007) ± 0.004 | 0.005 ± 0.002 | (0.008) ± 0.004 |
| D-melibiose | 0.000 ± 0.003 | (0.001) ± 0.003 | 0.023 ± 0.010 | 0.036 ± 0.004 | 0.002 ± 0.001 |
| β-methyl-D-glucoside | (0.026) ± 0.006 | (0.040) ± 0.005 | (0.013) ± 0.007 | 0.004 ± 0.002 | (0.021) ± 0.004 |
| D-salicin | 0.033 ± 0.032 | 0.002± 0.001 | (0.021) ± 0.005 | (0.009) ± 0.003 | 0.068 ± 0.006 |
| N-acetyl-D-glucosamine | (0.022) ± 0.007 | (0.030) ± 0.003 | (0.004) ± 0.002 | 0.004 ± 0.002 | (0.010) ± 0.006 |
| N-acetyl-D-mannosamine | (0.027) ± 0.002 | (0.039) ± 0.005 | (0.010) ± 0.005 | 0.006 ± 0.001 | (0.020) ± 0.001 |
| N-acetyl-D-galactosamine | (0.029) ± 0.001 | (0.041) ± 0.004 | 0.003 ±0 | 0.003 ± 0.001 | (0.023) ± 0.001 |
| N-acetyl-neuraminic acid | (0.056) ± 0.001 | (0.071) ± 0.005 | (0.036) ± 0.008 | (0.015) ± 0.001 | (0.042) ± 0.003 |
| α-D-glucose | (0.025) ± 0.002 | (0.029) ± 0.004 | 0.005 ± 0.001 | 0.010 ± 0.001 | (0.017) ± 0.002 |
| D-mannose | (0.022) ± 0.002 | (0.030) ± 0.004 | 0.010 ± 0.006 | 0.013 ± 0.003 | (0.015) ± 0.002 |
| D-fructose | 0.040 ± 0.012 | 0.048 ± 0.003 | 0.072 ± 0.014 | 0.085 ± 0.015 | 0.032 ± 0.001 |
| D-galactose | (0.016) ± 0.003 | (0.018) ± 0.004 | (0.047) ± 0.015 | (0.038) ± 0.001 | (0.029) ± 0.005 |
| 3-methyl-glucose | 0.002 ± 0.006 | (0.032) ± 0.003 | (0.015) ± 0.007 | (0.017) ± 0.001 | (0.006) ± 0.004 |
| D-fucose | (0.014) ± 0.002 | (0.005) ± 0.003 | (0.036) ± 0.006 | (0.045) ± 0.003 | (0.019) ± 0.003 |
| L-fucose | 0.085 ± 0.004 | 0.068 ± 0.001 | 0.105 ± 0.004 | 0.108 ± 0.005 | 0.087 ± 0.002 |
| L-rhamnose | (0.008) ± 0.002 | (0.011) ±0 | 0.040 ± 0.006 | 0.040 ± 0.001 | (0.005) ±0 |
| Inosine | (0.054) ± 0.004 | (0.061) ± 0.009 | (0.028) ± 0.009 | (0.010) ± 0.002 | (0.051) ± 0.003 |
| D-sorbitol | (0.027) ± 0.002 | (0.015) ± 0.008 | 0.004 ± 0.001 | 0.006 ± 0.001 | (0.014) ± 0.002 |
| D-mannitol | (0.026) ± 0.004 | (0.029) ± 0.004 | 0.012 ± 0.009 | 0.006 ± 0.003 | (0.018) ± 0.002 |
| D-arabitol | (0.028) ± 0.003 | 0.004 ± 0.002 | 0.009 ± 0.006 | 0.005 ± 0.001 | (0.022) ± 0.001 |
| myo-inositol | (0.025) ± 0.003 | (0.025) ± 0.007 | 0.014 ± 0.008 | 0.006 ± 0.004 | (0.015) ± 0.003 |
| glycerol | 0.275 ± 0.027 | 0.391 ± 0.019 | 0.138 ± 0.015 | 0.022 ± 0.006 | 0.422 ± 0.013 |
| D-glucose-6-phosphate | (0.017) ± 0.005 | (0.033) ± 0.005 | 0.005 ±0 | 0.014 ± 0.001 | (0.020) ± 0.002 |
| D-fructose-6-phosphate | 0.111 ± 0.009 | 0.108 ±0.003 | 0.127 ± 0.009 | 0.148 ± 0.007 | 0.095 ± 0.005 |
| D-aspartic acid | (0.009) ± 0.002 | (0.003) ± 0.002 | (0.019) ± 0.007 | (0.007) ± 0.003 | (0.019) ± 0.001 |
| D-serine | (0.029) ± 0.003 | (0.005) ± 0.004 | (0.013) ± 0.007 | (0.009) ± 0.003 | (0.013) ± 0.006 |
| Gelatin | (0.038) ± 0.006 | (0.038) ± 0.004 | (0.018) ± 0.010 | (0.011) ± 0.002 | (0.031) ± 0.003 |
| Glycyl-L-proline | (0.007) ± 0.003 | (0.003) ± 0.006 | 0.019 ± 0.012 | 0.003 ± 0.001 | (0.001) ± 0.002 |
| L-alanine | (0.031) ± 0.003 | (0.042) ± 0.004 | (0.012) ± 0.007 | (0.009) ± 0.001 | (0.028) ± 0.002 |
| ±L-arginine | (0.014) ± 0.002 | (0.021) ± 0.008 | (0.006) ± 0.003 | (0.002) ± 0.001 | (0.017) ± 0.006 |
| L-aspartic acid | (0.001) ± 0.004 | 0.040 ± 0.010 | 0.019 ± 0.009 | (0.006) ± 0.001 | (0.003) ± 0.002 |
| L-glutamic acid | 0.028 ± 0.007 | 0.104 ± 0.010 | 0.052 ± 0.012 | (0.004) ± 0.001 | 0.022 ± 0.005 |
| ±L-histidine | (0.028) ± 0.003 | (0.027) ±0.005 | 0.005 ±0 | 0.011 ± 0.001 | (0.024) ± 0.006 |
| L-pyroglutamic acid | (0.017) ± 0.004 | 0.006 ± 0.004 | 0.017 ± 0.004 | (0.006) ± 0.002 | (0.014) ± 0.005 |
| L-serine | (0.025) ± 0.002 | (0.014) ± 0.009 | (0.005) ± 0.004 | (0.010) ± 0.002 | (0.027) ± 0.004 |
| Pectin | (0.035) ± 0.007 | (0.030) ± 0.001 | 0.003 ± 0.001 | 0.003 ± 0.001 | (0.034) ± 0.010 |
| D-galacturonic acid | 0.029 ± 0.013 | 0.052 ± 0.003 | 0.084 ± 0.011 | 0.098 ± 0.012 | 0.025 ± 0.002 |
| L-galactonic acid lactone | (0.065) ± 0.005 | (0.058) ± 0.001 | (0.031) ± 0.010 | (0.017) ± 0.002 | (0.053) ± 0.001 |
| D-gluconic acid | (0.021) ± 0.002 | (0.022) ± 0.003 | 0.012 ± 0.008 | (0.009) ± 0.002 | (0.015) ± 0.001 |
| D-glucuronic acid | 0.045 ± 0.004 | 0.099 ± 0.005 | 0.128 ± 0.011 | 0.141 ± 0.013 | 0.057 ± 0.005 |
| Glucuronamide | 0.221 ± 0.008 | 0.226 ± 0.007 | 0.254 ± 0.019 | 0.268 ± 0.003 | 0.213 ± 0.002 |
| Muric acid | (0.029) ± 0.005 | (0.019) ± 0.002 | 0.001 ±0 | (0.010) ± 0.003 | (0.029) ± 0.001 |
| Quinic acid | (0.034) ± 0.008 | (0.017) ± 0.006 | 0.013 ±0.004 | (0.012) ± 0.004 | (0.021) ± 0.006 |
| D-saccharic acid | (0.025) ± 0.004 | (0.037) ± 0.004 | (0.014) ± 0.006 | (0.014) ± 0.004 | (0.028) ± 0.005 |
| p-Hydroxy-phenylacetate | (0.080) ± 0.010 | (0.093) ± 0.003 | (0.046) ± 0.009 | (0.026) ± 0.002 | (0.079) ± 0.004 |
| Methyl pyruvate | (0.028) ± 0.008 | 0.028 ± 0.013 | 0.020 ± 0.017 | (0.002) ± 0.001 | (0.018) ± 0.004 |
| D-Lactic acid methyl ester | (0.004) ± 0.002 | 0.030 ± 0.001 | 0.034 ± 0.008 | 0.026 ± 0.001 | (0.002) ± 0.001 |
| L-Lactic acid | (0.018) ± 0.011 | 0.022 ± 0.006 | 0.020 ± 0.007 | (0.019) ± 0.001 | 0.017 ± 0.007 |
| Citric acid | 0.044 ± 0.014 | 0.079 ± 0.010 | 0.076 ± 0.017 | 0.012 ± 0.004 | 0.047 ± 0.003 |
| α-Keto-glutaric acid | 0.096 ± 0.004 | 0.184 ± 0.005 | 0.230 ± 0.014 | 0.158 ± 0.009 | 0.116 ± 0.015 |
| D-malic acid | (0.028) ± 0.003 | (0.003) ± 0.001 | 0.015 ± 0.003 | (0.007) ±0 | (0.029) ± 0.002 |
| L-malic acid | (0.017) ± 0.006 | 0.0260 ± 0.003 | 0.050 ± 0.002 | 0.045 ± 0.018 | (0.002) ± 0.004 |
| Bromo-succinic acid | (0.016) ± 0.004 | 0.013 ± 0.001 | 0.022 ± 0.004 | (0.006) ± 0.001 | (0.013) ± 0.009 |
| Tween 40 | 0.045 ± 0.009 | 0.289 ± 0.029 | 0.020 ± 0.004 | 0.018 ± 0.002 | 0.269 ± 0.005 |
| γ-Amino-butryic acid | (0.017) ± 0.006 | (0.003) ± 0.001 | 0.022 ± 0.011 | (0.008) ± 0.005 | (0.012) ± 0.002 |
| α-hydro-butryic acid | (0.065) ± 0.005 | (0.074) ± 0.003 | (0.035) ± 0.011 | (0.020) ± 0.001 | (0.058) ± 0.008 |
| β-hydro-D,L-butryric acid | (0.009) ± 0.004 | 0.0460 ±0 | 0.040 ± 0.011 | (0.007) ± 0.002 | (0.004) ± 0.002 |
| α-Keto-butyric acid | 0.023 ± 0.009 | 0.0440 ± 0.017 | 0.069 ± 0.016 | 0.092 ± 0.007 | 0.030 ± 0.006 |
| Acetoacetic acid | 0.156 ± 0.002 | 0.212 ± 0.005 | 0.214 ± 0.016 | 0.194 ± 0.013 | 0.145 ± 0.014 |
| Propionic acid | 0.019 ± 0.002 | 0.090 ± 0.009 | 0.043 ± 0.003 | 0.019 ± 0.003 | 0.032 ± 0.006 |
| Acetic acid | 0.165 ± 0.023 | 0.361 ± 0.006 | 0.183 ± 0.002 | 0.045 ± 0.008 | 0.173 ± 0.016 |
| Formic acid | (0.036) ± 0.008 | (0.032) ± 0.005 | (0.020) ± 0.011 | (0.011) ± 0.002 | (0.027) ± 0.004 |

**Supplementary Table 4.** Variable Importance in Projection (VIP) scores and Kruskal-Wallis p-values from PLS-DA model of the metabolic phenotypes of Log, S4, S10, S20, and R6 cultures.

| Metabolite | VIP | Kruskal-Wallis p-value |
|---|---|---|
| Acetoacetic acid | 2.822 | 0.008 |
| β-hydroxy-D,L-butyric acid | 2.759 | 0.007 |
| Glycerol | 2.615 | 0.009 |
| Tween 40 | 2.599 | 0.007 |
| D-galactose[a] | 2.260 | 0.007 |
| Propionic acid | 2.074 | 0.012 |
| Acetic acid | 1.489 | 0.013 |
| D-trehalose | 1.448 | 0.016 |
| α-Ketoglutaric acid | 1.403 | 0.009 |
| Bromo-succinic acid[a] | 1.399 | 0.010 |
| D-fucose[a] | 1.292 | 0.013 |
| L-glutamic acid | 1.246 | 0.010 |
| Methyl pyruvate | 1.113 | 0.005 |
| Glucuronamide | 1.103 | 0.015 |
| D-arabitol | 1.082 | 0.007 |
| Citric acid | 1.024 | 0.053 |
| L-fucose | 1.011 | 0.010 |
| 3-methyl-glucose[a] | 1.009 | 0.012 |
| L-aspartic acid | 0.955 | 0.011 |
| Gentiobiose | 0.915 | 0.019 |
| D-glucuronic acid | 0.887 | 0.011 |
| D-fructose-6-phosphate | 0.834 | 0.011 |
| D-lactic acid methyl ester | 0.807 | 0.010 |
| D-salicin[a] | 0.778 | 0.046 |
| L-lactic acid | 0.778 | 0.021 |
| L-histidine | 0.752 | 0.005 |
| D-melibiose | 0.730 | 0.010 |
| α-D-glucose | 0.703 | 0.006 |
| L-malic acid | 0.671 | 0.008 |
| α-keto-butyric acid | 0.668 | 0.031 |
| Pectin[a] | 0.666 | 0.010 |
| L-pyroglutamic acid | 0.648 | 0.005 |
| D-glucose-6-phosphate | 0.644 | 0.007 |
| D-mannose | 0.636 | 0.007 |
| L-rhamnose | 0.620 | 0.007 |
| Dextrin | 0.608 | 0.011 |
| D-fructose | 0.566 | 0.021 |
| D-galacturonic acid | 0.535 | 0.014 |
| Quinic acid[a] | 0.455 | 0.005 |

[a] Induced abiotic reduction of tetrazolium dye

**Supplementary Table 5. Replicate numbers, sequencing depth, and quality control parameters for RNA-seq of BCG transcriptome before, during, and after starvation.** L = log growth in normal medium; S4, S10, S20 = days of growth in PBS (nutrient deprivation); R6 = days of resuscitation in normal medium.

| Sample | Treatment | Biological Replicate | Technical Replicate 1 | Technical Replicate 2 | Coverage (% mapped) | rRNA (%) | 3' to 5' ratio |
|---|---|---|---|---|---|---|---|
| | | | Number of Reads | | | | |
| L1 | Exponential growth | 1 | 1204645 | 1205127 | 99.4 | 0.2 | 1.07 |
| L2 | | 2 | 1395678 | 1401660 | 99.5 | 0.5 | 1.05 |
| L3 | | 3 | 1985455 | 1987693 | 99.2 | 0.2 | 1.06 |
| S4_1 | Starvation 4 days | 1 | 1528656 | 1532367 | 87.3 | 2.6 | 1.05 |
| S4_2 | | 2 | 1190809 | 1191191 | 92.9 | 0.6 | 1.02 |
| S4_3 | | 3 | 1577450 | 1583833 | 92.4 | 0.7 | 1.06 |
| S10_1 | Starvation 10 days | 1 | 1448874 | 1450220 | 95.0 | 1.4 | 0.92 |
| S10_2 | | 2 | 1519684 | 1522886 | 93.4 | 11.6 | 0.98 |
| S10_3 | | 3 | 1002837 | 1003414 | 94.5 | 6.7 | 0.97 |
| S20_1 | Starvation 20 days | 1 | 1241005 | 1245825 | 88.5 | 11.2 | 0.96 |
| S20_2 | | 2 | 1626951 | 1628710 | 94.6 | 7.5 | 0.97 |
| S20_3 | | 3 | 1304831 | 1307200 | 85.5 | 7.0 | 0.97 |
| R6_1 | Resuscitation 6 days after 20 days starvation | 1 | 1203096 | 1205831 | 98.8 | 1.7 | 1.09 |
| R6_2 | | 2 | 1143052 | 1146801 | 98.9 | 2.3 | 1.08 |
| R6_3 | | 3 | 1387610 | 1391396 | 90.7 | 20.5 | 1.11 |
| Average | | | 1384042 | 1386944 | 94.0 | 5.0 | 1.02 |

**Supplementary Table 6. Primers for qPCR.**

| Gene | Direction | Sequence | Melting Temperature (ºC) |
|---|---|---|---|
| *relA* | F | ATTGCCACCAGAAACACCGA | 91.50 |
| | R | GGTTCCGGGCGATGTGATTA | |
| *fdxA* | F | AGTGAGTGCGTGGATGTGATG | 88.50 |
| | R | TGGTGTTGATCGTCGGGTAGA | |
| *hspX* | F | GACGAGATGAAAGAGGGGCG | 90.00 |
| | R | GTCGTCCTCGTCAGCACCTA | |
| *ethA* | F | CGAGGCCGACGTTCTACTTAT | 90.00 |
| | R | GCGACTTCGACACTGGTTGC | |
| *cyp135A1* | F | GTGAAGCGGCGGAAAATCTC | 90.50 |
| | R | CACCCCAAACCCACAGAGTT | |
| *cyp128* | F | TCCCGAGAACAGTGCATTCC | 90.50 |
| | R | TCCGGGTTGATTTCGCTTGT | |
| *sigA* [14] | F | CGATGAGCCGGTAAAACGC | 91.00 |
| | R | GAGCCACTAGCGGACTTCGC | |

**Supplementary Discussion**

Together with our previous reports of tRNA landscape remodeling and codon usage adaptation in hypoxia and starvation-induced NRP in mycobacteria[15,16], our integrated multi-omic observations from the transcriptomic, proteomic, metabolomic and phenotypic studies detailed here suggest broadly divergent, and often opposing, molecular responses at all levels, that surprisingly lead to similar multi-drug resistant phenotypes. This divergence provides unique insights into the adaptative biochemistry of NRP and thereby opens new areas for rationally designing modalities to overcome multi-drug resistance. For instance, under the classical Wayne model, hypoxia induces resistance to first-line drugs such as isoniazid but confers susceptibility to metronidazole[17]. However, this susceptibility to metronidazole is lost in nutrient starvation models[18] and in combined stress models[19]. Our results (**Figs. 3-5**), suggest a mechanism in starvation-induced NRP that begins with an accumulation of ketone bodies AcAc and BHB, causing intracellular acidification and inducing ROS-generating pathways – the latter likely mediated by cytochrome proteins (CYP) (**Figs. 3b, 3f, 5a**). CYP activity is a well-established source of ROS[20] with strong correlations to cellular basal ROS production, and BHB metabolism.

One possible explanation for the observed toxicity of $H_2O_2$ to starvation induced NRP is the uncoupling of CYP activity from BHB metabolism and ROS generation. The first suggestion of this activity arose from the observation that exposure to millimolar concentrations of $H_2O_2$ proved to be bactericidal to S10 and S20, but not to Log or R6 BCG (**Fig. 5d**). This seemingly paradoxical behavior can be explained as $H_2O_2$-facilitated killing through CYP inactivation[21]. While $H_2O_2$ can serve as an electron donor to drive CYP activity by generating Cpd 0 directly from the ferric enzyme through the peroxidise shunt, the CYP heme group and catalytic cysteine residues are exquisitely susceptible to $H_2O_2$ inactivation by Fe-mediated reduction to generate locally damaging hydroxyl radicals or ferryl-oxo species (i.e., Fenton chemistry)[12]. This is especially plausible as all the preconditions for Haber-Weiss and Bray-Gorin catalysis, except one, were met within the starved mycobacteria: acidic pH, ferric and ferrous ions, and superoxide generation from CYP activity[22]. The missing element is elevated $H_2O_2$. This is consistent with

down-regulation of genes coding for $H_2O_2$-producing enzymes in starved bacilli and with the lack of adaptive changes in catalase activity and *katG* expression (**Figs. 5b,c**) and accumulation of BHB upon $H_2O_2$ exposures (**Fig. 5g**). These findings provide further evidence that previous reports of increased endogenous ROS levels as the underlying antibiotic bactericidal mechanism could be expanded by considering NRP as pharmacologically distinct from exponentially growing cells due to their altered metabolic capabilities[23,24].

**Supplementary References**


1 Stallings, C. L. *et al.* CarD is an essential regulator of rRNA transcription required for Mycobacterium tuberculosis persistence. *Cell* **138**, 146-159 (2009). https://doi.org:10.1016/j.cell.2009.04.041

2 Bochner, B. R. New technologies to assess genotype-phenotype relationships. *Nat Rev Genet* **4**, 309-314 (2003). https://doi.org:10.1038/nrg1046

3 Bochner, B. R. Global phenotypic characterization of bacteria. *FEMS Microbiol Rev* **33**, 191-205 (2009). https://doi.org:10.1111/j.1574-6976.2008.00149.x

4 DiChiara, J. M. *et al.* Multiple small RNAs identified in Mycobacterium bovis BCG are also expressed in Mycobacterium tuberculosis and Mycobacterium smegmatis. *Nucleic Acids Res* **38**, 4067-4078 (2010). https://doi.org:10.1093/nar/gkq101

5 Wegscheid, B. & Hartmann, R. K. In vivo and in vitro investigation of bacterial type B RNase P interaction with tRNA 3'-CCA. *Nucleic Acids Res* **35**, 2060-2073 (2007). https://doi.org:10.1093/nar/gkm005

6 Keiler, K. C. Biology of trans-translation. *Annu Rev Microbiol* **62**, 133-151 (2008). https://doi.org:10.1146/annurev.micro.62.081307.162948

7 Hartkoorn, R. C. *et al.* Genome-wide definition of the SigF regulon in Mycobacterium tuberculosis. *J Bacteriol* **194**, 2001-2009 (2012). https://doi.org:10.1128/JB.06692-11

8 Theriault, M. E. *et al.* Iron limitation in M. tuberculosis has broad impact on central carbon metabolism. *Commun Biol* **5**, 685 (2022). https://doi.org:10.1038/s42003-022-03650-z

9 Chao, A., Sieminski, P. J., Owens, C. P. & Goulding, C. W. Iron Acquisition in Mycobacterium tuberculosis. *Chem Rev* **119**, 1193-1220 (2019). https://doi.org:10.1021/acs.chemrev.8b00285

10 Boelaert, J. R., Vandecasteele, S. J., Appelberg, R. & Gordeuk, V. R. The effect of the host's iron status on tuberculosis. *J Infect Dis* **195**, 1745-1753 (2007). https://doi.org:10.1086/518040

11 Rodriguez, G. M., Sharma, N., Biswas, A. & Sharma, N. The Iron Response of Mycobacterium tuberculosis and Its Implications for Tuberculosis Pathogenesis and Novel Therapeutics. *Front Cell Infect Microbiol* **12**, 876667 (2022). https://doi.org:10.3389/fcimb.2022.876667

12 Munro, A. W., Girvan, H. M., Mason, A. E., Dunford, A. J. & McLean, K. J. What makes a P450 tick? *Trends Biochem Sci* **38**, 140-150 (2013). https://doi.org:10.1016/j.tibs.2012.11.006

13 Hajem Bataineh, O. P., Andreja Bakac. pH-induced mechanistic changeover from hydroxyl radicals to iron(iv) in the Fenton reaction. *Chemical Science* (2012).

14 Cappelli, G. *et al.* Profiling of Mycobacterium tuberculosis gene expression during human macrophage infection: upregulation of the alternative sigma factor G, a group of transcriptional regulators, and proteins with unknown function. *Res Microbiol* **157**, 445-455 (2006). https://doi.org:S0923-2508(05)00276-7 [pii]

10.1016/j.resmic.2005.10.007

15 Chionh, Y. H. *et al.* tRNA-mediated codon-biased translation in mycobacterial hypoxic persistence. *Nat Commun* **7**, 13302 (2016). https://doi.org:10.1038/ncomms13302

16 Hu, J. F. *et al.* Quantitative mapping of the cellular small RNA landscape with AQRNA-seq. *Nature biotechnology* **39**, 978-988 (2021). https://doi.org:10.1038/s41587-021-00874-y

17 Wayne, L. G. In Vitro Model of Hypoxically Induced Nonreplicating Persistence of Mycobacterium tuberculosis. *Methods Mol Med* **54**, 247-269 (2001). https://doi.org:10.1385/1-59259-147-7:247

18 Betts, J. C., Lukey, P. T., Robb, L. C., McAdam, R. A. & Duncan, K. Evaluation of a nutrient starvation model of Mycobacterium tuberculosis persistence by gene and protein expression profiling. *Mol Microbiol* **43**, 717-731 (2002). https://doi.org:2779 [pii]

19 Gibson, S. E. R., Harrison, J. & Cox, J. A. G. Drug Susceptibility Screening Using In Vitro Models of Hypoxic Non-Replicating Persistent Mycobacteria. *Methods Mol Biol* **2314**, 247-260 (2021). https://doi.org:10.1007/978-1-0716-1460-0_10

20 Denisov, I. G., Makris, T. M., Sligar, S. G. & Schlichting, I. Structure and chemistry of cytochrome P450. *Chem Rev* **105**, 2253-2277 (2005). https://doi.org:10.1021/cr0307143

21 Karuzina, II, Zgoda, V. G., Kuznetsova, G. P., Samenkova, N. F. & Archakov, A. I. Heme and apoprotein modification of cytochrome P450 2B4 during its oxidative inactivation in monooxygenase reconstituted system. *Free Radic Biol Med* **26**, 620-632 (1999). https://doi.org:10.1016/s0891-5849(98)00252-4

22 Bataineh, H., Pestovsky, O. & Bakac, A. pH-induced mechanistic changeover from hydroxyl radicals to iron(iv) in the Fenton reaction. *Chemical Science* **3**, 1594-1599 (2012).

23 Brynildsen, M. P., Winkler, J. A., Spina, C. S., MacDonald, I. C. & Collins, J. J. Potentiating antibacterial activity by predictably enhancing endogenous microbial ROS production. *Nature biotechnology* **31**, 160-165 (2013). https://doi.org:10.1038/nbt.2458

24 Grant, S. S., Kaufmann, B. B., Chand, N. S., Haseley, N. & Hung, D. T. Eradication of bacterial persisters with antibiotic-generated hydroxyl radicals. *Proc Natl Acad Sci U S A* **109**, 12147-12152 (2012). https://doi.org:10.1073/pnas.1203735109
