# Supplementary material for: Facile metabolic reprogramming distinguishes mycobacterial adaptation to hypoxia and starvation: ketosis drives starvation-induced persistence in M. bovis BCG
Source: Commun Biol. 2024 Jul 16;7:866. doi: 10.1038/s42003-024-06562-2 (PMC11250799; doi:10.1038/s42003-024-06562-2)
Supplement: Supplementary file 2 — Description of Additional Supplementary Materials [file 42003_2024_6562_MOESM2_ESM.pdf]

Description of Additional Supplementary Files

File name: Supplementary Data 1

Description: Proteomic analysis of starved BCG

File name: Supplementary Data 2

Description: RNA-seq data for Starved BCG

File name: Supplementary Data 3

Description: Metabolomic analysis of starved and hypoxic BCG

File name: Supplementary Data 4

Description: The Source data for all figures and supplementary figures
